# Brain functional connectivity difference in the complete network of an entire village: the role of social network size and embeddedness

**DOI:** 10.1038/s41598-017-04904-1

**Published:** 2017-06-30

**Authors:** Won-tak Joo, Seyul Kwak, Yoosik Youm, Jeanyung Chey

**Affiliations:** 1 Department of Sociology, University of Wisconsin-Madison, Wisconsin, USA; 2 Department of Psychology, Seoul National University, Gwanak-ro 1, Gwanak-gu, Seoul, South Korea; 3 Department of Sociology, Yonsei University, Yonsei-ro 50, Seodaemun-gu, Seoul, South Korea

## Abstract

Social networks are known to protect cognitive function in old age. For the first time, this study examines how social network size and social network embeddedness measured by k-core score are associated with functional connectivity in the brain using the complete social network of an entire village. According to the results, social network size has both positive and negative associations with functional connectivity; showing no meaningful pattern relative to distance among brain regions. However, older adults deeply embedded in the complete network tend to maintain functional connectivity between long-distance regions even after controlling for other covariates such as age, gender, education, and Mini-Mental State Examination score. Network Based Statistics (NBS) also revealed strong and consistent evidence that social network embeddedness has component-level associations with functional connectivity among brain regions, especially between inferior prefrontal and occipital/parietal lobes.

## Introduction

The association between social networks and cognitive health has been investigated in epidemiology studies. The large body of literature indicates that interaction in large social networks^1, 2^ or participation in various social activities^3, 4^ is protective of cognitive function in later life. In particular, cognitive stimuli from socio-economic environments, such as education or social relationships, could promote more effective cognitive networks, accommodate more brain lesions without cognitive decline, and, subsequently, help maintain a better cognitive function at older ages^5, 6^. While recent studies using functional magnetic resonance imaging (fMRI) showed that the main cause of cognitive impairment in later life results from the destruction in functional connectivity among brain regions of interest (ROIs)^7, 8^, Marques and colleagues reported that higher levels of education provide more efficient brain functional networks and alleviate the negative effects of aging^9^. According to their analyses, years of education are positively associated with functional connectivity among distant brain ROIs, especially between anterior and posterior regions, which could help the brain to efficiently mobilize its segregated regions. This study aims to expand the postulate of Marques and colleagues to the effects of social networks: how could social networks be related to the change in brain functional connectivity?

In many previous studies, social network measurements were mainly based on self-reports about the social relationships or the amount of social support of respondents. In this study, we collected the social interaction information from all older adults residing in a rural township in South Korea, allowing us to examine the global structure of social networks in an entire village^10^. Considering both the results from resting-state fMRI scan on 64 healthy older adults without brain pathology and social network data, we investigated how brain functional connectivity is associated with social network size and embeddedness within the township.

## Results

### Description of the participants

Table 1 presents the descriptive statistics of the participants and pairwise correlation coefficients among variables. Out of 64 participants, the sample includes 42 (66%) females of an average age of 71.39 and with an average Mini-Mental State Examination (MMSE) score of 26.50. Considering that it takes six years to complete elementary school in South Korea, the average years of education (6.14) is quite low, but accurately reflects rural Korean older population’s educational characteristics.

**Table 1:** Descriptive statistics of the participants (n = 64).

Variable	Mean	SD	Min	Max	Pairwise correlation				
					[1]	[2]	[3]	[4]	[5]
[1] Female	0.66	0.48	0	1	—				
[2] MMSE score	26.50	2.77	16	30	−0.179	—			
[3] Age	71.39	6.39	59	84	−0.116	−0.272*	—		
[4] Years of education	6.14	3.68	0	20	−0.477*	0.516*	−0.175	—	
[5] Social network size	6.22	5.43	0	28	−0.123	0.105	0.072	−0.094	—
[6] Social network embeddedness (continuous)	3.73	2.23	0	7	−0.042	0.009	−0.013	−0.135	0.846*
[7] Social network embeddedness (low = 0, high = 1)	0.44	0.50	0	1	−0.091	0.115	−0.134	−0.086	0.765*

Note: *p < 0.05.

**Figure 1:**
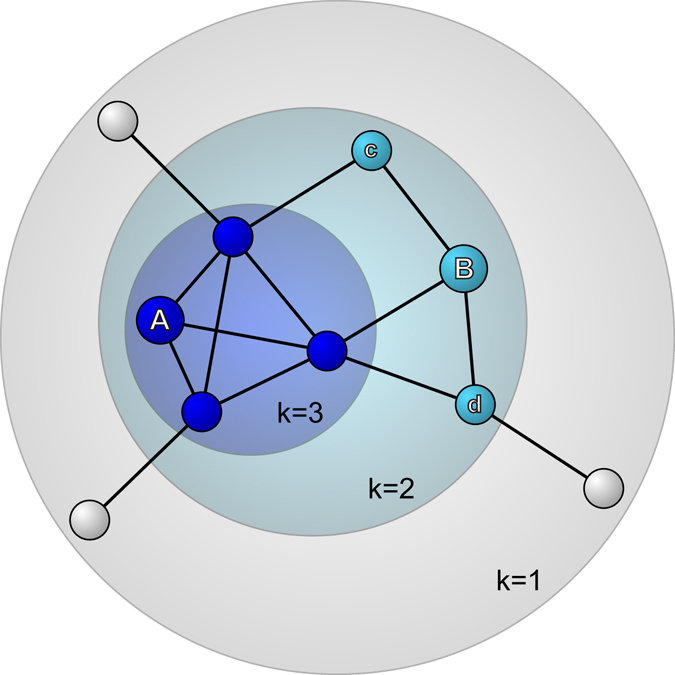
A hypothetical network graph of 10 people. Social network embeddedness was measured by *k*-core score, the highest value of *k* among *k*-core groups to which people belonged. A *k*-core group consists of people who have at least *k* social connections with group members. Even though A and B’s social network sizes are the same (3), only A can be a member of the 3-core group and has a higher k-core score than B (3 vs. 2).

Two social network indices were assessed using the complete network of Township K in South Korea (details about social network data construction are described in the methods section). First, *social network size* was measured by the number of social connections the participants had in the complete network. Second, *social network embeddedness* was measured by *k*-core score^11^. A *k*-core group is composed of people who have at least *k* social connections with others in the same group. Since one can belong to several nested *k*-core groups from low *k* to high *k*, he or she takes the highest value of *k* as his or her *k*-core score. For example, A in Fig. 1 belongs to three kinds of *k*-core groups (a 1-core group composed of all 10 people in the graph, a 2-core group of 7 people, and a 3-core group of 4 people), thus A’s *k*-core score is 3, corresponding to the maximum of *k*. On the other hand, B’s *k*-core score is 2 since B is not a member of the 3-core group. Even though B has the same size of social networks as A (3), B cannot hold 3 social connections when c and d (having only two social connections in the 2-core group) are excluded from the 3-core group. In this case, A is more likely than B to belong to the core of the social groups, and thus to be more deeply embedded in people’s networks. Since social network embeddedness is originally a discreet measure, we considered both a continuous and a binary form (split at the median, > 3) when performing the analyses.

**Figure 2:**
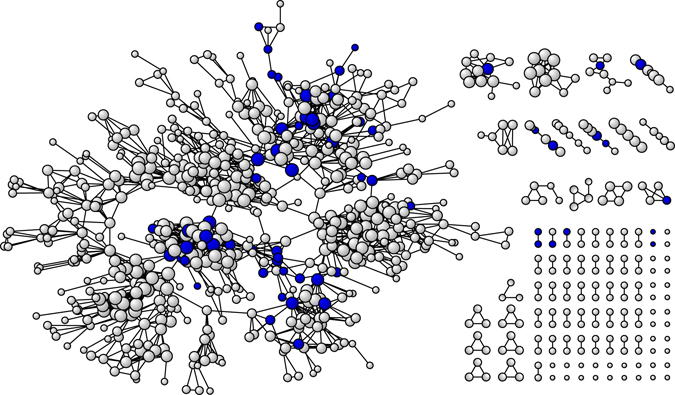
A complete network of 830 individuals in Township K. A circle stands for each individual, and a line for social connection between individuals. Bigger size represents higher score of social network embeddedness. Final samples for this study (n = 64) are colored in blue.

Figure 2 illustrates the complete network of Township K, which consists of 830 residents including 64 participants of this study (colored blue). As seen in the figure, participants with higher scores of social network embeddedness (circles of bigger size) are connected to communities of more individuals and the dense social connections could possibly foster more consistent and stronger social stimuli on the brain via emotional support and cognitive challenges. The correlation between social network size and social network embeddedness are very high (0.846), suggesting that individuals who were more deeply embedded in the township networks were usually maintaining social connections with more township residents.

In the next sections, we examined the associations between functional connectivity and individual characteristics (including social networks) using two types of statistical tests. First, we considered functional connectivity within each pair of ROIs separately as a predicted variable of the generalized linear model (GLM) in order to test *pair-level* associations. Second, we tested if there was a collective change in functional connectivity of ROIs using Network Based Statistics (NBS)^12^. NBS detects components of functionally connected ROIs from pair-level analyses and tests the significance of *component-level* associations by comparing the results with those from simulated brain functional networks (statistical analyses are presented in detail in the methods section). All the analyses were performed using three different sets of ROIs from the Anatomical Automatic Labeling (AAL) atlas^13^, the Harvard-Oxford (HO) probabilistic atlas^14^, and the Dosenbach atlas^15^. In this paper, the results from the AAL atlas were mainly discussed, and those from HO and Dosenbach atlases were presented in the supplement online.

### Pair-level associations with functional connectivity

**Figure 3:**
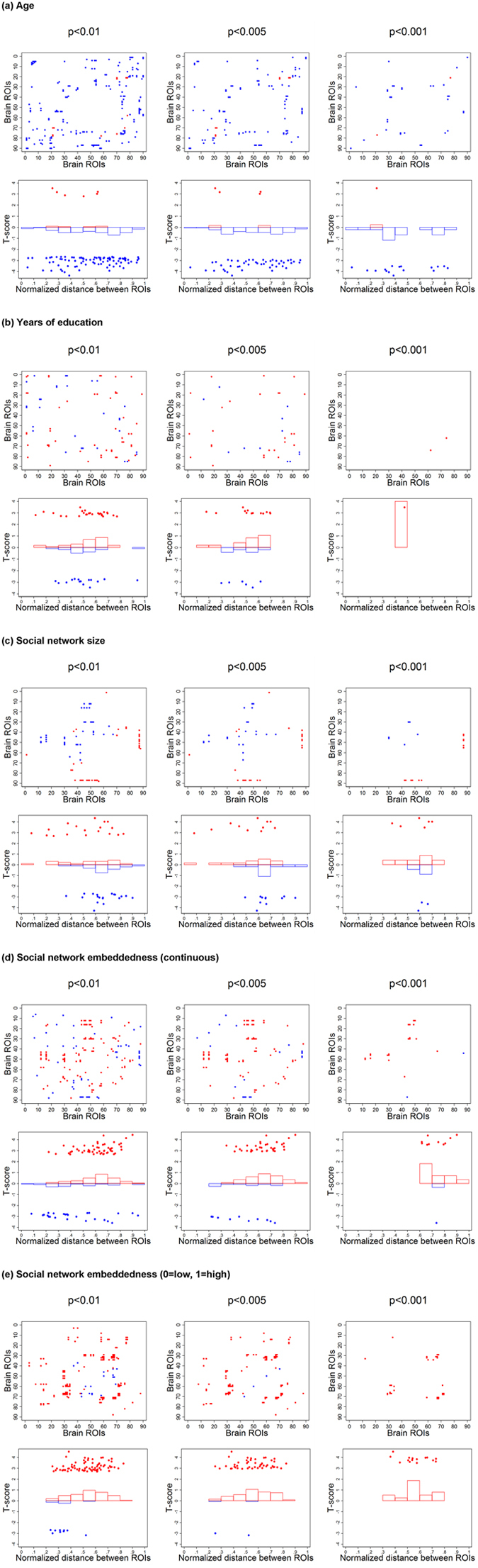
Pair-level associations between functional connectivity and individual characteristics. Blue/red dots represent negative/positive associations significant at the thresholds of p < 0.01, p < 0.005, or p < 0.001. Scatterplots in the first row are matrix representations of associations with functional connectivity among 90 by 90 brain ROIs. Scatterplots and histograms in the second row illustrate the associations by distance between brain ROIs. (**a**) Age was negatively associated with overall functional connectivity. (**b**) Years of education and (**c**) social network size showed associations with functional connectivity among only a few ROI-pairs at the level of p < 0.001, which did not show any trend in distance between ROIs. (**d**) Social network embeddedness (continuous) and (**e**) social network embeddedness (low = 0, high = 1) showed positive associations with functional connectivity, especially between long-distance ROIs in the brain.

Figure 3 illustrates pair-level differences in brain connectivity by age, years of education, social network size, and social embeddedness. As seen in Fig. 3a, old age was negatively associated with overall functional connectivity among ROI-pairs, whose distribution by distance was slightly left-skewed. However, positive correlations between age and functional connectivity were found in a few pairs. The associations with years of education in Fig. 3b did not show any distinctive patterns by distance between brain regions, and only one pair remained with a strong threshold of p < 0.001. As for social network size in Fig. 3c, we observed both positive and negative associations with several ROI-pairs. While the distance between ROI-pairs was evenly distributed, we observed positive correlations with brain connectivity centered on the left middle temporal gyrus at the level of p < 0.001. Figure 3d shows that the majority of associations between functional connectivity and social network embeddedness were positive, and mainly distributed in long-distance regions between frontal and occipital lobes. Similar patterns are observed in Fig. 3e that participants with high social network embeddedness had stronger functional connectivity among distal ROIs than participants with lower-than-median embeddedness scores. When considering a binary form, however, ROIs were identified from pairs between frontal and parietal lobes.

The results from HO and Dosenbach atlases were similar with those from the AAL atlas (the results were presented in Supplementary Figures S6 and S7). Age had strong negative associations with functional connectivity between many long-distance ROIs, and strong positive associations with a small number of short-distance ROIs. Negatively-associated ROI-pairs had clearer left-skewed distribution by distance than those from the AAL atlas. Social network size showed no distinctive pattern, whereas social network embeddedness had strong positive associations with long-distance ROIs when using both atlases. Interestingly, years of education had strong positive associations with short- and middle-ranged ROIs when using HO and Dosenbach atlases than the AAL atlas. The majority of those pairs were between temporal and occipital lobes.

### Component-level associations with functional connectivity

**Figure 4:**
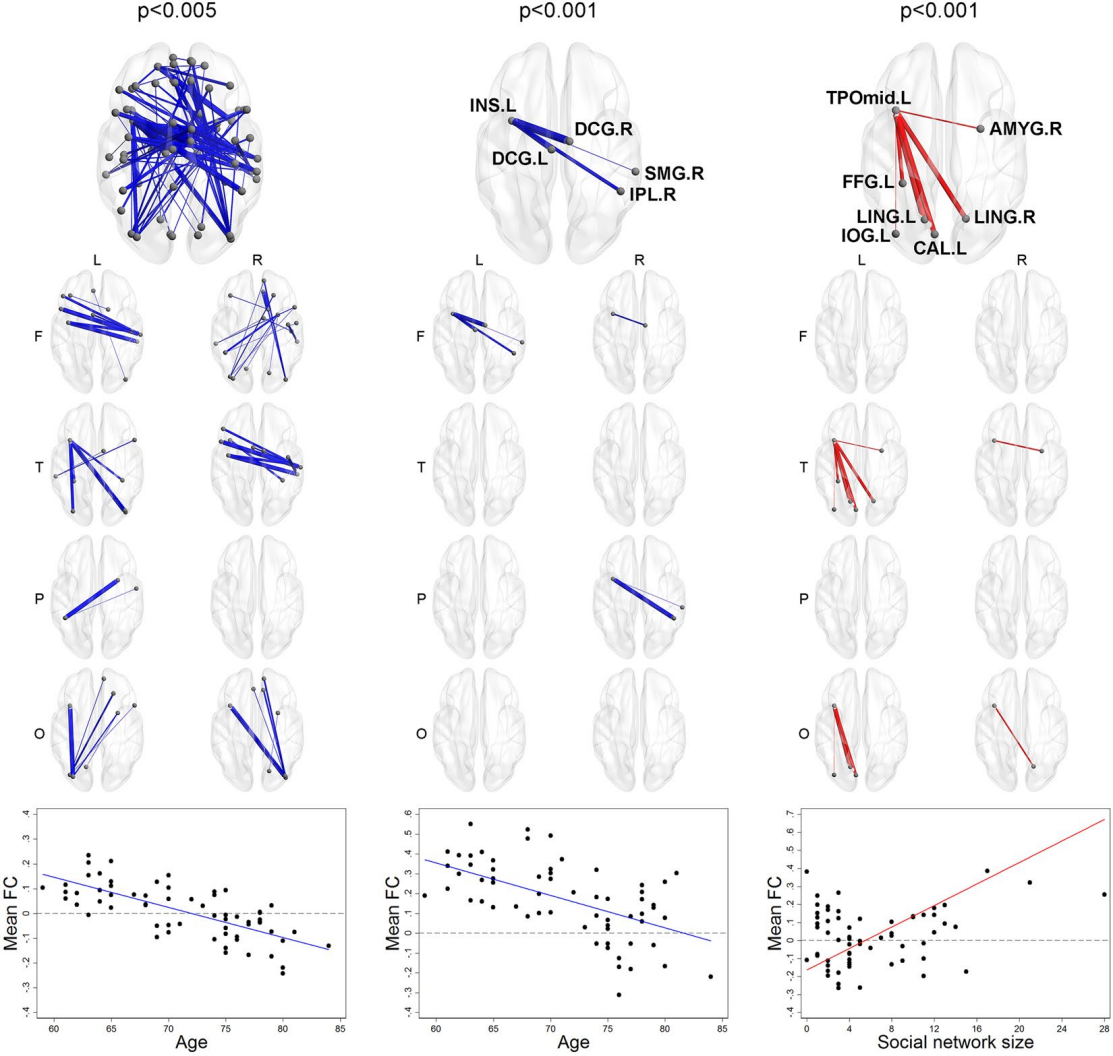
Component-level associations between functional connectivity and age/social network size. A brain figure for the whole component and eight separate figures for the regional connectivity from left/right frontal/temporal/parietal/occipital lobes are presented for each threshold. The largest component negatively associated with age at the thresholds of p < 0.01 or p < 0.005 had significantly larger extent and intensity than simulated networks, but not at the level of p < 0.001. As for social network size, the component only at the level of p < 0.001 had significantly larger intensity than simulated networks. Mean functional connectivity among the ROIs in each component was lower for those with higher age, and higher for those with the bigger social networks after controlling for other covariates.

Table 2 presents the NBS test results aimed at uncovering any component-level difference by age, years of education, social network size, and social embeddedness (the lists of ROIs in components detected by NBS are presented in Supplementary Table S1 to S4, and the network graphs based on the Kamada-Kawai algorithm^16^ are in Supplementary Figures S1 to S3). The components negatively associated with age at all thresholds had significantly larger extent (the number of ROI-pairs) and intensity (the extent weighted by the strength of associations) than simulated networks at the level of p_NBS_ < 0.05, except for intensity at a threshold of p < 0.005 and for extent at a threshold of p < 0.001. Mean functional connectivity of components detected by NBS showed negative associations with age after controlling for gender, MMSE score, social network size, and social network embeddedness. As for years of education, we could not observe any significant component-level association from NBS. Regarding social network size, the component positively associated at a threshold of p < 0.001 and had significantly larger extent and intensity than simulated networks. As shown in Fig. 4, the components included the ROI-pairs between the temporal lobe (right amygdala, left fusiform gyrus, and left middle temporal gyrus) and the occipital lobe (calcarine fissure, lingual gyrus, and left inferior occipital gyrus). Mean functional connectivity of components detected by NBS at a threshold of p < 0.001 also showed a positive association with social network size after controlling for other individual characteristics.

**Table 2:** Network Based Statistics (NBS) for testing component-level associations between functional connectivity and individual characteristics (n = 64).

Variables	Association	Statistics	Component threshold					
			p < 0.01		p < 0.005		p < 0.001	
			Size	(p_NBS_)	Size	(p_NBS_)	Size	(p_NBS_)
Age	−	Extent	86	(0.011)	28	(0.031)	4	(0.083)
		Intensity	35.9	(0.011)	8.0	(0.057)	2.1	(0.044)
Years of education	+	Extent	19	(0.229)	8	(0.228)	1	(0.628)
		Intensity	4.7	(0.331)	0.9	(0.590)	0.0	(0.625)
Social network size	+	Extent	12	(0.347)	8	(0.225)	6	(0.042)
		Intensity	9.4	(0.156)	6.7	(0.081)	2.5	(0.037)
Social network embeddedness (continuous)	+	Extent	48	(0.051)	32	(0.024)	9	(0.017)
		Intensity	24.7	(0.030)	14.5	(0.020)	3.7	(0.016)
Social network embeddedness (low = 0, high = 1)	+	Extent	73	(0.016)	47	(0.010)	12	(0.010)
		Intensity	36.6	(0.011)	20.9	(0.010)	3.8	(0.017)

Note: From each set of ROI-pairs selected by pair-level tests with three different thresholds of p < 0.01, p < 0.005, and p < 0.001, NBS detects the largest ROI-component based on the extent (the number of ROI-pairs) or intensity (the extent weighted by the strength of associations), and calculates p-values (p_NBS_) from the rate of cases where empirical brain networks have larger extent or intensity than 5,000 simulated networks. All the analyses were performed with covariates of gender, MMSE score, age, years of education, social network size, and social network embeddedness. The continuous form of social network embeddedness was used when testing the hypotheses for other major predictors.

**Figure 5:**
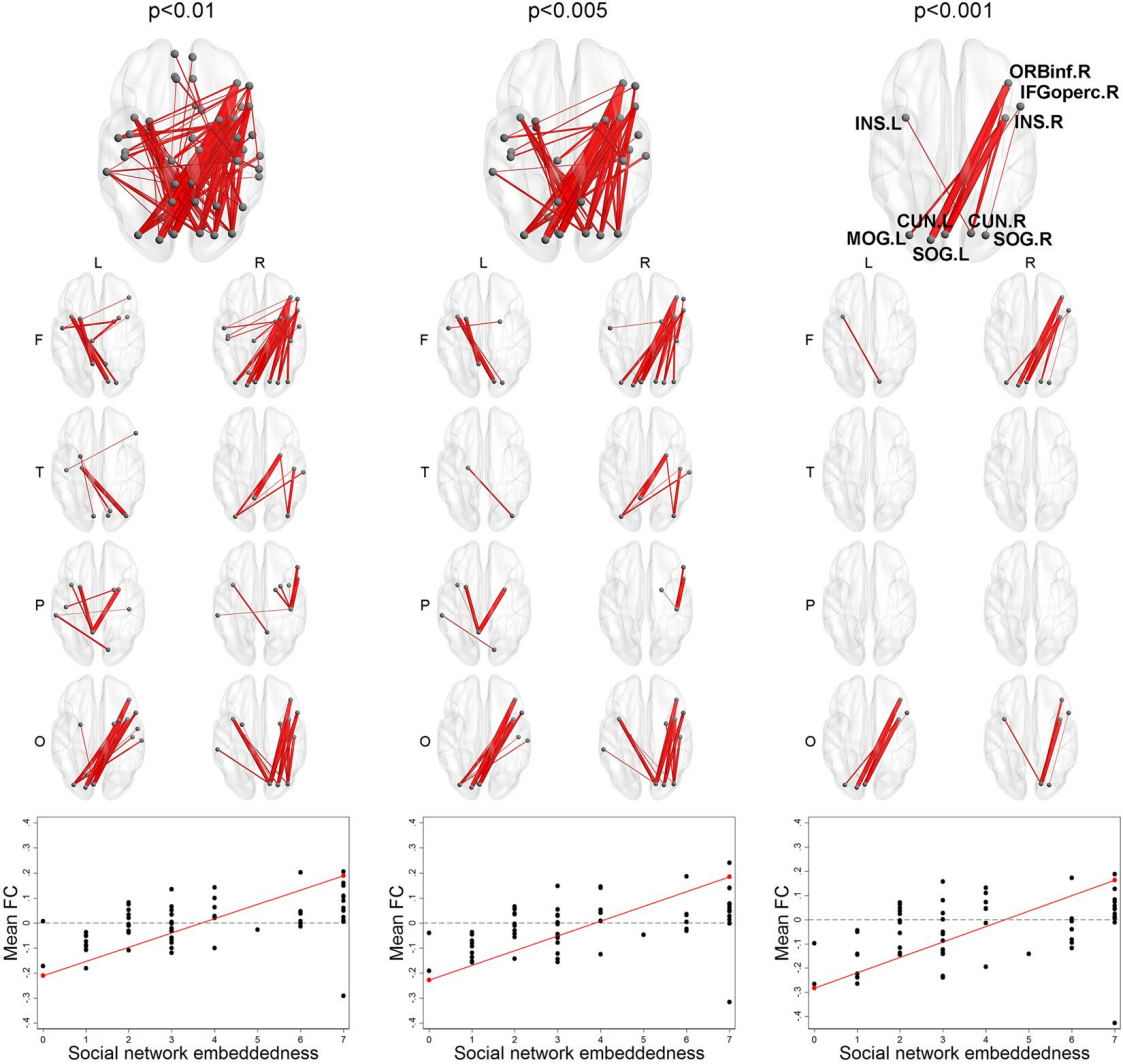
Component-level associations between functional connectivity and social network embeddedness (continuous). A brain figure for the whole component and eight separate figures for the regional connectivity from left/right frontal/temporal/parietal/occipital lobes are presented for each threshold. The largest component positively associated with social network embeddedness at the thresholds of p < 0.01, p < 0.005, or p < 0.001 had significantly larger extent and intensity than simulated networks. Mean functional connectivity among the ROIs in each component was higher for those with higher social network embeddedness.

**Figure 6:**
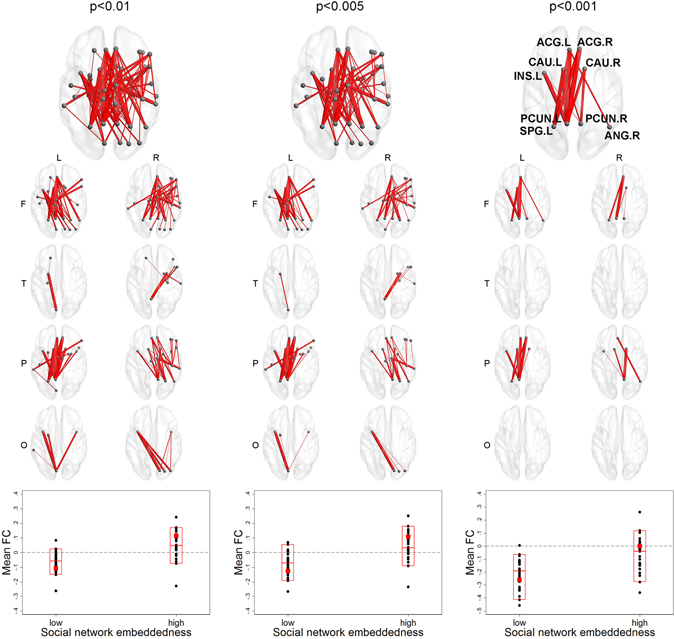
Component-level associations between functional connectivity and social network embeddedness (low = 0, high = 1). A brain figure for the whole component and eight separate figures for the regional connectivity from left/right frontal/temporal/parietal/occipital lobes are presented for each threshold. The largest component positively associated with social network embeddedness at the thresholds of p < 0.01, p < 0.005, or p < 0.001 had significantly larger extent and intensity than simulated networks. Mean functional connectivity among the ROIs in each component was higher for those with high social network embeddedness than low social network embeddedness.

Social network embeddedness showed significant evidence for component-level associations from NBS. When considering a continuous form, the largest components whose functional connectivity between ROI-pairs were positively associated with social network embeddedness at all three thresholds of p < 0.01, p < 0.005, and p < 0.001 had significantly larger extent and intensity than simulated networks. As seen in Fig. 5, The largest component at the level of p < 0.001 was composed of ROI-pairs from the right frontal lobe (right inferior frontal gyrus, right insula) to the occipital lobe (cuneus, superior occipital gyrus, and middle occipital gyrus). When considering a binary form, the largest components from all thresholds were also significantly larger than simulated networks. In Fig. 6, you can find that the largest component at the level of p < 0.001 was centered on the left insula, the cingulate gyri, the caudate nucleus, and the parietal lobe (precuneus, left superior parietal gyrus). Mean functional connectivity of components detected by NBS at all thresholds showed positive associations with social network embeddedness after controlling for gender, MMSE score, years of education, and social network size (see Supplementary Table S5). When considering further models after excluding outliers^17^, the associations between mean functional connectivity and age, social network size, and social network embeddedness showed the same directions as the original models (presented in Supplementary Table S6, Figures S4, and S5). Additionally, we tested the interaction effects of age with education, social network size, or social network embeddedness, but no significant results were observed (presented in Supplementary Table S7).

According to the results from component-level analyses using the HO atlas (see Supplementary Tables S8, S12–14, and Figures S8–13), the components negatively associated with age at all thresholds had significantly larger extent and intensity than simulated networks. Unlike the results from the AAL atlas, however, years of education had significant and positive component-level associations with functional connectivity, especially between temporal (right superior temporal gyrus, left temporal occipital fusiform cortex) and occipital lobes (left lingual gyrus, intracalcarine cortex, cuneal cortex, supracalcarine cortex). As for social network size, no component was detected from component-level analyses. Regarding social network embeddedness, a binary form had strong and positive component-level associations with ROI-pairs between the right inferior frontal gyrus and the occipital lobe (right lateral occipital cortex), and the cingulate gyri and parietal (left superior parietal lobule, precuneous cortex)/occipital lobes (right lateral occipital cortex). Although a continuous form of social network embeddedness was marginally significant at thresholds of p < 0.01 and p < 0.005, strong component-level associations were mainly identified from ROI-pairs between right frontal and occipital lobes. Component-level analyses using the Dosenbach atlas, however, did not identify any components associated with major predictors at all thresholds (see Supplementary Table S10). The interaction effects between age and other major predictors were not significant when considering HO or Dosenbach atlases (see Supplementary Tables S9 and S11).

## Discussion

Functional connectivity of the brain is often illustrated as a *small world* network, where i) proximate brain regions are functionally clustered, and ii) inter-cluster connectivity between several ROIs enables those clusters to efficiently synchronize^18^. Long-distance connectivity in the brain, usually linking segregated clusters of brain regions, are considered to play important roles in making the brain a small but efficient world. Studies on Alzheimer’s disease patients showed that the small world structure is destroyed not because of disruption in local connectivity but in long-distance connectivity between anterior (prefrontal lobe) and posterior regions (parietal and occipital lobes)^19–21^. Similarly, aging population studies also demonstrate that old age is correlated with a decrease in long-distance connectivity in default mode networks and attention networks^7^, resulting in less efficient brain function^22^ with more locally-integrated but globally-segregated functional networks^8^. Although this study did not directly adopt the indices for small world, the results provide robust evidence that social networks, especially social network embeddedness in the global network of the community, is positively associated with functional connectivity between anterior-posterior regions.

Pair-level tests in this study show that the old age is correlated with the decrease in functional connectivity especially between long-distance regions, which are coherent with the results from the previous studies^7, 9^. While several models provided positive correlations with functional connectivity between temporal and occipital lobes, the results from pair-level tests on education level and social network size are inconsistent when using different brain atlases. Social network embeddedness, meanwhile, has consistent and positive associations with functional connectivity between long-distance ROI-pairs from all pair-level tests using different thresholds and brain atlases. Since the decrease in connectivity by age was mostly found between left and right parietal/temporal lobes, social network embeddedness may not directly make up for the disadvantage of aging. However, if connectivity between posterior-anterior regions provides alternative routes for functional networks among brain regions, it could indirectly compensate the process of cognitive aging.

The results from component-level tests provided robust evidence of associations between functional connectivity and social network embeddedness, especially between inferior prefrontal cortex and occipital/parietal lobes, when using AAL and HO atlases. Moreover, positive associations with distal brain regions were consistently observed in the two different atlases. On the other hand, all the component-level analyses based on the Dosenbach atlas did not provide any significant results. In order to be identified as a component from component-level tests, ROI-pairs should i) have strong associations with the predictor in pair-level tests, and ii) share some common ROIs as junctions of those pairs. While pair-level tests using the Dosenbach atlas selected many long-distance ROI-pairs, they were failed to be identified as a component because they had no common junctions but only neighboring ROIs. Since the Dosenbach atlas was the most detailed atlas among the three we used, the brain might be split into so small regions to have common points among ROI-pairs. Additionally, the different results from atlases could also arise from partial volume effects. In order to address this problem, we conducted denoising procedure implemented in the Conn toolbox, thus we partly regressed out the effect induced in the white matter and cerebrospinal fluid tissue area. Since these procedures were done before extraction of ROI time-series values and connectivity calculation, we expect partial volume confounds to be moderately adjusted. Despite no supporting evidence from the Dosenbach atlas, the results from other atlases were enough to implicate the importance of social network embeddedness in function connectivity of older adults.

The advantage of well-organized social networks can be explained in two ways. First, our results imply that social networks could provide cognitive stimuli similar to those from the educational experience, and these cognitive stimuli help promote functional connectivity among distant ROIs. As shown in the study by Marques and colleagues, chronic stimulations from educational experiences are related to stronger functional connectivity among post-anterior ROIs, which could compensate the localized functional networks of aged people^9^. Similar results were observed in the resting-state positron emission tomography (PET) study on Korean urban samples: participants with higher education had higher small-worldness scores of functional connectivity, which were measured by the ratio of clustering coefficient to average path length among ROIs^23^. While the analyses of education using different brain atlases provided inconsistent results, social networks had strong and robust associations with long-distance functional connectivity of the brain. Considering that 71.46% (576 out of 814) of participants responded that they graduated from only elementary school or did not have any experience of formal education in the first wave of this project^10^, little variation in education might undermine its effects on the brain. On the other hand, well-organized social networks are likely more essential in generating cognitive activities in urban socio-economic contexts. Second, social networks could be related to functional connectivity via structural changes in the brain^24^. Studies have reported that chronic exposure to elevated glucocorticoid caused by life stress induce inflammation and cardiovascular diseases^25^, potentially related to the volume decrease of hippocampus and prefrontal cortex^26^, and white matter integrity of the brain^27^. Considering that social support from networks alleviates emotional distress from life events^28^, our results could partly derive from disadvantaged brain structure of those with poor social networks.

The main strength of this study comes from the extensive information about social relationships among all older residents in the township. Social network embeddedness can only be measured within a complete network of all people of interest. We found that the major contribution of the social network does not depend on the network size, but depends on the network embeddedness. Older adults with deeply embedded positions take advantage of three distinctive types of resources. First, since they belong to a very cohesive core group, all their social friends tend to share the same values, attitudes, and behaviors, thus, they will provide various types of support in a *consistent* and *coordinated* way: friends in the group with high *k*-core score will coordinate their actions to help other friends in the group and can provide consistent valuable help. Groups with high *k*-core score also can provide help and share activities in a more *robust* way. Since all members tend to be friends with each other, even when a couple of members are sick or become hostile, the whole friendship circle can be relatively easily maintained and thus, still provide the same valuable support to each other. Lastly, since they are more likely to act together as a single group, they maintain their cognitive function to meet the challenge of coordinating multiple actions from different people. We believe that those benefits of social network embeddedness could help older adults cumulate better social support and more cognitive stimuli on the brain, which may consequently activate functional connectivity among brain ROIs.

One of limitations in this study is its cross-sectional design. It is possible that inefficient functional connectivity in the brain can cause the decrease in cognitive function, and consequently interfere with social participation and socializing with neighbors. Despite the limitation, this study is, as far as we know, the first study on the association between brain functional connectivity and social network characteristics using the global network of a whole community. Future studies using longitudinal data with large observations will be able to complement the findings of this study in the following ways:

i) The relationship between social networks and brain structure should be investigated. In another study using the same data presented in this study examined how social network size correlates with regional gray matter volume^29^. The results showed that the size of inward social networks – number of people who designated the respondent as their network member – was significantly associated with brain regions involving social information processing (lateral orbitofrontal cortex, amygdala and temporo-parietal junction area) while whole-brain exploratory analysis showed association in medial occipital region. Moreover, higher episodic memory function mediated the relation between brain structure and inward social network size. These results implicate that larger social brain volume leads to larger social connectedness, which in turn, also benefits whole brain health in general. According to accumulating studies, stronger functional integration within amygdala network may indicate the capacity to process social information that supports larger social network^30^. As partly observed in this research, the size of social network was associated with ventrolateral amygdala network that encompasses from temporal pole to inferior temporal cortex. On the other hand, significant functional connectivity effects across diffuse and long-distance connectivity may indicate white matter integrity influenced by inflammatory response vulnerability^27^. In this study, however, it is hard to pin down the source of the effect on regional functional connectivity strength. It is assumed that, especially in older adults, socio-affective function and cognitive function bi-directionally influences each other^31^. If the brain structure promotes the formation of functional connectivity, a longitudinal pathway from social networks - brain structure - functional connectivity can be identified.
ii) The relationship between various social network properties and brain features should be studied. Individuals’ positions and potentials in social networks can be measured in diverse ways. For example, in the study of social networks and brain structure mentioned before, outward and inward social networks were separately measured. We could not consider the direction of social networks in this study since we added data on social relationships among network members to obtain more variation in *k*-core scores, and for which no information on direction was available. When we considered inward and outward social network size instead of our measures for network size, however, NBS still did not detect any significant component-level difference by network size of both directions, whereas the component-level associations with social network embeddedness were robust after controlling for any measures of social network size. These findings do not rule out the possibility of network size effects on functional connectivity since they are premature to determine the causal paths among network size and embeddedness. Future studies will be able to clarify the co-evolving process of social networks and the brain.
iii) The final goal of social network-brain studies is to identify how these processes result in cognitive function and health of older adults. In an on-going research using 4-year data from the residents in the same township with this study, the positive associations between social network embedded and MMSE scores were found. While cross-sectional associations between MMSE scores and functional connectivity were not observed in this study, it is unclear how the present functional connectivity will affect the change in MMSE scores and the onset of brain diseases in the future. Longitudinal data on functional connectivity will make it possible to reveal the mechanism in which social networks are related to cognitive function and health via functional connectivity in the brain.

## Methods

### Participants

The study samples were from the 3^rd^ wave of Korean Social Life, Health and Aging Project (KSHAP). KSHAP is a community-based cohort study on health and social networks of older adults from ten villages in Township K, Ganghwa-gun, Incheon, South Korea. The 1^st^ wave investigation was performed from December 2011 to March 2012 targeting all residents aged 60 or older and their spouses in Township K. The survey and health examination were conducted in the respondents’ home or community centers, which resulted in a response rate of 94.65% (814/860). In the 3^rd^ wave, 591 older adults participated in the follow-up survey in February 2014, and 195 from three villages completed further screening tests for preclinical neurocognitive disorders in January and February 2015. The criteria for significant cognitive impairment were as follows: 1) participants who scored below 1.5 SD in Mini-Mental State Examination for Dementia Screening (MMSE-DS)^32^, 2) those who were in the 5 percentile on Long-term Memory Recall Index or Working Memory Index in Elderly Memory Scale^33^ based on age and education specified norm, and 3) those who had significant cognitive or behavioral changes in a past year from the semi-structured interview of Clinical Dementia Rating (CDR)^34^. Sixty-eight participants who passed the screening tests underwent a functional MRI scan at the Seoul National University Brain Imaging Center, and 3 were excluded due to excessive scan movement, neurological abnormality, and diffuse signal confounds, respectively. After excluding one more participant who did not complete the social network survey in the 3^rd^ wave, 64 observations of brain functional images and social networks were examined in the final analyses. The study was approved by and performed in accordance with the relevant guidelines and regulations by the Institutional Review Board of Yonsei University, and all participants provided written informed consent for the research procedure.

### Brain functional connectivity

Resting-state fMRI data were acquired on a 3T Siemens Trio scanner. During the scan, participants were instructed to rest quietly with their eyes open and not to fall asleep. We acquired 300 contiguous EPI functional images (TR = 2000 ms, TE = 30 ms, FOV = 240 × 240 mm, FA = 79°, voxel size 3 × 3 × 3 mm, gap = 1 mm, acquisition time = 10 minutes). In order to acquire high spatial resolution, cerebellar regions were excluded in the acquisition. T1-weighted magnetic prepared rapid gradient echo (MPRAGE) image were acquired (Sagittal slices, slice thickness 1 mm, TR = 2300 ms, TE = 2.36 ms, FOV = 256 × 256 mm, FA = 9°, voxel size 1 × 1 × 1 mm³).

Image preprocessing and denoising was performed using the SPM12 software (Welcome Department of Imaging Neuroscience, Institute of Neurology, London, UK) with the conn toolbox 15 (http://www.nitrc.org/projects/conn) for functional connectivity analysis. Functional images were corrected for slice time and motion. EPI images were warped into MNI standard space. Images were smoothed with a Gaussian kernel of 8 mm³ full-width half-maximum. Three participants who had consecutive images of head movement estimates above 5 mm were discarded. In addition, the Artifact Detection Tools (https://www.nitrc.org/projects/artifact_detect/) was used to identify motion and signal intensity outlier images. Images with global mean intensity Z-value > 9 and movement > 2 mm were identified as outlier images (Outlier scans average = 4.98, SD = 7.45). Estimated motion parameters and outlier images were used as nuisance covariates in the time-series linear regression. T1-weighted images were segmented into gray matter, white matter and cerebrospinal fluid and warped into MNI standard space. Signals within white matter and CSF mask were regressed out to exclude non-gray matter BOLD signal. Band-pass temporal filtering (0.008–0.09) was applied to exclude physiological noise signal. For each subject, mean time-series were extracted by averaging all voxels composing each region for each time point from 90 cortical, subcortical ROIs excluding cerebellar regions from the Anatomical Automatic Labeling (AAL) atlas^13^. Pearson correlation coefficients were calculated between each pair of regions and transformed to Fisher’s Z scores. Finally, 64 individual functional connectivity matrices containing (90 × (90–1))/2 = 4,005 pairwise functional connectivity values were constructed. For the robustness check, we performed the supplementary analyses using connectivity matrices from the Harvard-Oxford probabilistic atlas (105 ROIs excluding cerebellum)^14^ and the Dosenbach atlas (142 ROIs excluding cerebellum)^15^.

### Social networks

Social network variables were created with the complete network from the 3^rd^ wave of KSHAP. The respondents enumerated their social network members (a spouse if any, up to five people who most often discussed important concerns over the last 12 months, and one very important person if any) with the information about real names, gender, residence, frequency of contact with network members (day/year), and frequency of contact between each pair of network members^10^. In order to combine these respondent-centered networks as a complete network of Township K, the same people who appeared in different respondents’ networks were identified based on the following criteria: 1) those who were not married with the respondents of the 3^rd^ wave survey and living outside of Township K were excluded, 2) at least two out of three Korean characters in their names matched, 3) their gender was the same, 4) their age difference was less than five years, and 5) their addresses belonged to the same village. From this process, we identified 830 unique individuals in Township K. Social connections among those individuals were assumed to exist if they had as frequent as the median (182.5 days a year) or more frequent contact. Finally, we constructed the complete network of 830 nodes and 1,879 undirected social connections. Social network size and social network embeddedness were calculated using *Pajek*.

### Statistical analysis

Two types of statistical analyses were performed. First, we examined the relationship between social networks and the *pair-level* difference in the brain. We examined 4,005 separate generalized linear models (GLMs) predicting brain connectivity between each pair of 90 ROIs with four major predictors (age, years of education, social network size, social network embeddedness) and covariates (gender, MMSE score). We considered both a continuous and a binary form (split at the median, > 3) of social network embeddedness, while only the continuous form was included in the models when testing other major predictors. Additionally, we identified ROI-pairs whose functional connectivity had strong associations with each major predictor based on three different thresholds (p < 0.01, p < 0.005, and p < 0.001), and explored the distribution by Euclidian distance between ROIs within each pair. The distance was divided by the maximum distance in the brain and normalized within a range from 0 to 1.

Second, we tested if social networks were associated with the *component-level* difference in connectivity using Network Based Statistics (NBS)^12^. Since pair-level tests only concern the relationship between each ROI-pair and predictors, it is hard to capture how these pairs collectively make a difference in the brain connectivity, and how big this difference is. NBS was proposed to provide statistics for a collective variation and perform a simulation-based test which strongly controls the family-wise error rate from mass-univariate analyses of brain ROIs. Instead of voxel-wise statistics based on a cluster of physically neighboring voxels, NBS adopts a *network component* of functionally connected ROIs and test the hypotheses according to the following procedures. i) Network components are identified from ROI-pairs selected by pair-level tests. If you consider each selected ROI-pair as one connection, you could find ROI-components whose members are directly or indirectly connected within the component, and separate from the outside. Since we considered five major predictors (including both forms of social network embeddedness) and three different thresholds (p < 0.01, p < 0.005, and p < 0.001), we could identify 5 × 3 = 15 different sets of ROI-pairs and network components for each predictor and threshold. For example, if you get four ROI-pairs [(1, 2), (1, 4), (3, 4), (5, 6)] from pair-level tests on social network embeddedness with a threshold of p < 0.01, you can identify two components A (1, 2, 3, 4) and B (5, 6). ii) NBS detects the largest component based on the extent (the number of ROI-pairs) or intensity (the extent weighted by the strength of associations – the strength is measured by the difference between the T-statistic from the pair-level test and the critical value for the threshold) of components. In the previous example, let’s assume that the T-statistics for social network embedded from all four pair-level tests are the same as 4, and the critical value for p < 0.01 is 2.7 (57 degrees of freedom, two-tail test). Then, A’s extent is 3 [(1, 2), (1, 4), (3, 4)], and intensity is 3 × (4 − 2.7) = 3.9. Since B’s extent (1) and intensity (1.3) are smaller than A’s, A would be the largest component in this case. While NBS can identify different largest components when using two different criteria of extent and intensity, no such cases were found in this study. iii) NBS tests the hypotheses by comparing the extent (or intensity) of the largest component with simulated brain networks. Since we had two statistics (extent or intensity) for 15 sets of ROI-pairs and components, we reported 30 different results from NBS tests. P-values are calculated from the rate of cases where empirical brain networks have larger extent or intensity than simulated networks. In this study, we tested the hypotheses at the level of p < 0.05 using 5,000 simulated brain matrices. In order to differentiate p-values used in the component thresholds from those in NBS tests, we used the notation *p* for the threshold criterion and *p*_*NBS*_ for NBS tests. Components were visualized using BrainNet Viewer^35^. For a clearer understanding of connectivity structure, we presented i) a figure for the whole connectivity of each component and eight separate figures for the regional connectivity from left/right frontal/temporal/parietal/occipital lobes, and ii) additional network graphs based on the Kamada-Kawai algorithm^16^ in the supplement online. We also presented the scatterplots that showed bivariate distributions between mean functional connectivity of components and major predictors, and trend lines from OLS regression analyses predicting mean connectivity of components detected by NBS with the covariates. When considering a binary form of social network embeddedness, we presented red boxes with three horizontal bars representing the 5^th^, 50^th^, and 95^th^ percentiles of mean functional connectivity, and red dots for the predicted scores of mean functional connectivity from OLS regression analyses after controlling for other covariates. For alleviating the effects from influential observations, we excluded outliers and re-examined OLS regression models. Outliers were detected by |DFBETA|>2/$\sqrt{n}$ (in this study, 2/$\sqrt{n}$ = 2/$\sqrt{64}$ = 0.25)^17^. DFBETA is calculated by the difference between the coefficient from all observations and the coefficient excluding the *i*th observation, scaled by the standard error of the original coefficient.

## Electronic supplementary material

Supplementary Information
