# Supplementary material for: Brain functional connectivity difference in the complete network of an entire village: the role of social network size and embeddedness
Source: Sci Rep. 2017 Jun 30;7:4465. doi: 10.1038/s41598-017-04904-1 (PMC5493622; doi:10.1038/s41598-017-04904-1)
Supplement: Supplementary file 1 — Supplementary Information [file 41598_2017_4904_MOESM1_ESM.pdf]

Brain functional connectivity difference in the complete network of an entire village

: the role of social network size and embeddedness

Won-tak Joo[1], Seyul Kwak[2], Yoosik Youm[3*], Jeanyung Chey[2*]

[1] Department of Sociology, University of Wisconsin-Madison

[2] Department of Psychology, Seoul National University

[3] Department of Sociology, Yonsei University

* Corresponding authors:

Yoosik Youm (Department of Sociology, Yonsei University; Yonsei-ro 50, Seodaemun-gu, Seoul, South Korea; yoosik@yonsei.ac.kr; +82-2-2123-2431)

Jeanyung Chey (Department of Psychology, Seoul National University; Gwanak-ro 1, Gwanak-gu, Seoul, South Korea; jychey@snu.ac.kr; +82-2-880-6432)

Supplementary Tables

Table S1. Brain Regions Of Interest (ROIs) in functional network components associated with age detected by Network Based Statistics (NBS) (regions from the Anatomical Automatic Labeling atlas)

Brain ROIs				T	Component threshold		
Source		Target			p<0.01	p<0.005	p<0.001
1	Precentral gyrus (Left)	80	Heschl gyrus (Right)	-3.34	*	*	
1	Precentral gyrus (Left)	81	Superior temporal gyrus (Left)	-2.68	*		
1	Precentral gyrus (Left)	82	Superior temporal gyrus (Right)	-2.76	*		
1	Precentral gyrus (Left)	90	Inferior temporal gyrus (Right)	-3.52	*	*	
2	Precentral gyrus (Right)	90	Inferior temporal gyrus (Right)	-3.36	*	*	
3	Superior frontal gyrus, dorsolateral (Left)	47	Lingual gyrus (Left)	-2.73	*		
3	Superior frontal gyrus, dorsolateral (Left)	64	Supramarginal gyrus (Right)	-2.75	*		
3	Superior frontal gyrus, dorsolateral (Left)	80	Heschl gyrus (Right)	-2.91	*		
4	Superior frontal gyrus, dorsolateral (Right)	51	Middle occipital gyrus (Left)	-2.82	*		
4	Superior frontal gyrus, dorsolateral (Right)	53	Inferior occipital gyrus (Left)	-3.10	*	*	
4	Superior frontal gyrus, dorsolateral (Right)	54	Inferior occipital gyrus (Right)	-2.85	*		
5	Superior frontal gyrus, orbital part (Left)	6	Superior frontal gyrus, orbital part (Right)	-2.74	*		
5	Superior frontal gyrus, orbital part (Left)	7	Middle frontal gyrus (Left)	-2.94	*		
5	Superior frontal gyrus, orbital part (Left)	8	Middle frontal gyrus (Right)	-3.23	*		
5	Superior frontal gyrus, orbital part (Left)	30	Insula (Right)	-3.88	*		
6	Superior frontal gyrus, orbital part (Right)	7	Middle frontal gyrus (Left)	-2.74	*		
6	Superior frontal gyrus, orbital part (Right)	72	Caudate nucleus (Right)	-2.68	*		
7	Middle frontal gyrus (Left)	86	Middle temporal gyrus (Right)	-2.81	*		
11	Inferior frontal gyrus, opercular part (Left)	46	Cuneus (Right)	-2.69	*		
11	Inferior frontal gyrus, opercular part (Left)	82	Superior temporal gyrus (Right)	-3.55	*	*	
12	Inferior frontal gyrus, opercular part (Right)	51	Middle occipital gyrus (Left)	-2.76	*		
12	Inferior frontal gyrus, opercular part (Right)	53	Inferior occipital gyrus (Left)	-2.93	*	*	
12	Inferior frontal gyrus, opercular part (Right)	85	Middle temporal gyrus (Left)	-3.05	*	*	

13	Inferior frontal gyrus, triangular part (Left)	82	Superior temporal gyrus (Right)	-3.28	*	*	
13	Inferior frontal gyrus, triangular part (Left)	86	Middle temporal gyrus (Right)	-2.82	*		
15	Inferior frontal gyrus, orbital part (Left)	72	Caudate nucleus (Right)	-3.01	*	*	
15	Inferior frontal gyrus, orbital part (Left)	78	Thalamus (Right)	-2.75	*		
18	Rolandic operculum (Right)	61	Inferior parietal, but supramarginal and angular gyri (Left)	-2.93	*	*	
18	Rolandic operculum (Right)	62	Inferior parietal, but supramarginal and angular gyri (Right)	-2.68	*		
19	Supplementary motor area (Left)	80	Heschl gyrus (Right)	-3.08	*	*	
19	Supplementary motor area (Left)	82	Superior temporal gyrus (Right)	-3.16	*	*	
19	Supplementary motor area (Left)	85	Middle temporal gyrus (Left)	-2.72	*		
19	Supplementary motor area (Left)	90	Inferior temporal gyrus (Right)	-2.83	*		
20	Supplementary motor area (Right)	79	Heschl gyrus (Left)	-2.81	*		
20	Supplementary motor area (Right)	80	Heschl gyrus (Right)	-3.03	*	*	
20	Supplementary motor area (Right)	85	Middle temporal gyrus (Left)	-2.93	*	*	
20	Supplementary motor area (Right)	90	Inferior temporal gyrus (Right)	-2.79	*		
23	Superior frontal gyrus, medial (Left)	54	Inferior occipital gyrus (Right)	-2.83	*		
24	Superior frontal gyrus, medial (Right)	53	Inferior occipital gyrus (Left)	-2.96	*	*	
24	Superior frontal gyrus, medial (Right)	54	Inferior occipital gyrus (Right)	-3.18	*	*	
25	Superior frontal gyrus, medial orbital (Left)	26	Superior frontal gyrus, medial orbital (Right)	-2.77	*		
25	Superior frontal gyrus, medial orbital (Left)	54	Inferior occipital gyrus (Right)	-2.70	*		
25	Superior frontal gyrus, medial orbital (Left)	72	Caudate nucleus (Right)	-2.88	*		
27	Gyrus rectus (Left)	54	Inferior occipital gyrus (Right)	-2.99	*	*	
28	Gyrus rectus (Right)	54	Inferior occipital gyrus (Right)	-3.18	*	*	
28	Gyrus rectus (Right)	72	Caudate nucleus (Right)	-3.92	*	*	
29	Insula (Left)	33	Median cingulate and paracingulate gyri (Left)	-4.05	*		*
29	Insula (Left)	34	Median cingulate and paracingulate gyri (Right)	-4.36	*		*
29	Insula (Left)	62	Inferior parietal, but supramarginal and angular gyri (Right)	-3.93	*		*
29	Insula (Left)	64	Supramarginal gyrus (Right)	-3.63	*		*
29	Insula (Left)	77	Thalamus (Left)	-3.16	*		

31	Anterior cingulate and paracingulate gyri (Left)	40	Parahippocampal gyrus (Right)	-3.53	*	
32	Anterior cingulate and paracingulate gyri (Right)	40	Parahippocampal gyrus (Right)	-3.55	*	
32	Anterior cingulate and paracingulate gyri (Right)	65	Angular gyrus (Left)	-3.03	*	
33	Median cingulate and paracingulate gyri (Left)	39	Parahippocampal gyrus (Left)	-2.72	*	
35	Posterior cingulate gyrus (Left)	74	Lenticular nucleus, putamen (Right)	-2.71	*	
39	Parahippocampal gyrus (Left)	73	Lenticular nucleus, putamen (Left)	-2.78	*	
39	Parahippocampal gyrus (Left)	75	Lenticular nucleus, pallidum (Left)	-3.90	*	
39	Parahippocampal gyrus (Left)	76	Lenticular nucleus, pallidum (Right)	-3.95	*	
40	Parahippocampal gyrus (Right)	88	Temporal pole: middle temporal gyrus (Right)	-2.70	*	
42	Amygdala (Right)	75	Lenticular nucleus, pallidum (Left)	-3.81	*	
43	Calcarine fissure and surrounding cortex (Left)	87	Temporal pole: middle temporal gyrus (Left)	-2.81	*	
44	Calcarine fissure and surrounding cortex (Right)	74	Lenticular nucleus, putamen (Right)	-2.94	*	*
44	Calcarine fissure and surrounding cortex (Right)	87	Temporal pole: middle temporal gyrus (Left)	-2.67	*	
45	Cuneus (Left)	74	Lenticular nucleus, putamen (Right)	-2.72	*	
47	Lingual gyrus (Left)	74	Lenticular nucleus, putamen (Right)	-2.94	*	*
51	Middle occipital gyrus (Left)	74	Lenticular nucleus, putamen (Right)	-3.04	*	*
51	Middle occipital gyrus (Left)	87	Temporal pole: middle temporal gyrus (Left)	-3.41	*	*
52	Middle occipital gyrus (Right)	87	Temporal pole: middle temporal gyrus (Left)	-3.23	*	*
52	Middle occipital gyrus (Right)	88	Temporal pole: middle temporal gyrus (Right)	-2.86	*	
53	Inferior occipital gyrus (Left)	87	Temporal pole: middle temporal gyrus (Left)	-2.75	*	
54	Inferior occipital gyrus (Right)	87	Temporal pole: middle temporal gyrus (Left)	-3.68	*	*
55	Fusiform gyrus (Left)	87	Temporal pole: middle temporal gyrus (Left)	-3.51	*	*
56	Fusiform gyrus (Right)	87	Temporal pole: middle temporal gyrus (Left)	-3.26	*	*
57	Postcentral gyrus (Left)	80	Heschl gyrus (Right)	-2.84	*	
57	Postcentral gyrus (Left)	90	Inferior temporal gyrus (Right)	-2.84	*	
58	Postcentral gyrus (Right)	90	Inferior temporal gyrus (Right)	-2.86	*	
61	Inferior parietal, but supramarginal and angular gyri (Left)	74	Lenticular nucleus, putamen (Right)	-3.31	*	*
62	Inferior parietal, but supramarginal and angular gyri (Right)	83	Temporal pole: superior temporal gyrus (Left)	-2.92	*	

64	Supramarginal gyrus (Right)	83	Temporal pole: superior temporal gyrus (Left)	-2.80	*
73	Lenticular nucleus, putamen (Left)	75	Lenticular nucleus, pallidum (Left)	-3.61	*
73	Lenticular nucleus, putamen (Left)	76	Lenticular nucleus, pallidum (Right)	-3.43	*
74	Lenticular nucleus, putamen (Right)	76	Lenticular nucleus, pallidum (Right)	-2.77	*
74	Lenticular nucleus, putamen (Right)	85	Middle temporal gyrus (Left)	-2.77	*
75	Lenticular nucleus, pallidum (Left)	76	Lenticular nucleus, pallidum (Right)	-3.12	*
79	Heschl gyrus (Left)	84	Temporal pole: superior temporal gyrus (Right)	-3.11	*

Table S2. Brain Regions Of Interest (ROIs) in functional network components associated with social network size detected by Network Based Statistics (NBS), component threshold of $p < 0.001$ (regions from the Anatomical Automatic Labeling atlas)

Brain ROIs				T
Source		Target		
42	Amygdala (Right)	87	Temporal pole: middle temporal gyrus (Left)	3.59
43	Calcarine fissure and surrounding cortex (Left)	87	Temporal pole: middle temporal gyrus (Left)	4.04
47	Lingual gyrus (Left)	87	Temporal pole: middle temporal gyrus (Left)	4.37
48	Lingual gyrus (Right)	87	Temporal pole: middle temporal gyrus (Left)	4.03
53	Inferior occipital gyrus (Left)	87	Temporal pole: middle temporal gyrus (Left)	3.48
55	Fusiform gyrus (Left)	87	Temporal pole: middle temporal gyrus (Left)	3.87

Table S3. Brain Regions Of Interest (ROIs) in functional network components associated with social network embeddedness (continuous) detected by Network Based Statistics (NBS) (regions from the Anatomical Automatic Labeling atlas)

Brain ROIs				T	Component threshold		
Source		Target			p<0.01	p<0.005	p<0.001
12	Inferior frontal gyrus, opercular part (Right)	43	Calcarine fissure and surrounding cortex (Left)	2.96	*	*	
12	Inferior frontal gyrus, opercular part (Right)	45	Cuneus (Left)	3.23	*	*	
12	Inferior frontal gyrus, opercular part (Right)	46	Cuneus (Right)	3.00	*	*	
12	Inferior frontal gyrus, opercular part (Right)	49	Superior occipital gyrus (Left)	3.57	*	*	*
12	Inferior frontal gyrus, opercular part (Right)	50	Superior occipital gyrus (Right)	3.54	*	*	*
12	Inferior frontal gyrus, opercular part (Right)	58	Postcentral gyrus (Right)	3.47	*	*	
14	Inferior frontal gyrus, triangular part (Right)	58	Postcentral gyrus (Right)	3.13	*	*	
14	Inferior frontal gyrus, triangular part (Right)	79	Heschl gyrus (Left)	2.80	*		
16	Inferior frontal gyrus, orbital part (Right)	17	Rolandic operculum (Left)	2.71	*		
16	Inferior frontal gyrus, orbital part (Right)	43	Calcarine fissure and surrounding cortex (Left)	3.10	*	*	
16	Inferior frontal gyrus, orbital part (Right)	45	Cuneus (Left)	4.14	*	*	*
16	Inferior frontal gyrus, orbital part (Right)	46	Cuneus (Right)	3.66	*	*	*
16	Inferior frontal gyrus, orbital part (Right)	47	Lingual gyrus (Left)	2.75	*		
16	Inferior frontal gyrus, orbital part (Right)	49	Superior occipital gyrus (Left)	4.45	*	*	*
16	Inferior frontal gyrus, orbital part (Right)	50	Superior occipital gyrus (Right)	3.36	*	*	
17	Rolandic operculum (Left)	76	Lenticular nucleus, pallidum (Right)	3.00	*	*	
18	Rolandic operculum (Right)	51	Middle occipital gyrus (Left)	2.68	*		
29	Insula (Left)	46	Cuneus (Right)	3.60	*	*	*
29	Insula (Left)	50	Superior occipital gyrus (Right)	3.00	*	*	
29	Insula (Left)	67	Precuneus (Left)	2.92	*	*	
29	Insula (Left)	68	Precuneus (Right)	2.89	*		
30	Insula (Right)	45	Cuneus (Left)	4.39	*	*	*
30	Insula (Right)	46	Cuneus (Right)	3.80	*	*	*
30	Insula (Right)	49	Superior occipital gyrus (Left)	3.18	*	*	
30	Insula (Right)	50	Superior occipital gyrus (Right)	3.30	*	*	

30	Insula (Right)	51	Middle occipital gyrus (Left)	3.78	*	*	*
30	Insula (Right)	52	Middle occipital gyrus (Right)	3.45	*	*	
30	Insula (Right)	58	Postcentral gyrus (Right)	2.67	*		
30	Insula (Right)	69	Paracentral lobule (Left)	2.68	*		
39	Parahippocampal gyrus (Left)	44	Calcarine fissure and surrounding cortex (Right)	2.84	*		
39	Parahippocampal gyrus (Left)	52	Middle occipital gyrus (Right)	2.94	*	*	
41	Amygdala (Left)	45	Cuneus (Left)	2.81	*		
41	Amygdala (Left)	46	Cuneus (Right)	2.79	*		
42	Amygdala (Right)	51	Middle occipital gyrus (Left)	3.26	*	*	
42	Amygdala (Right)	52	Middle occipital gyrus (Right)	3.11	*	*	
42	Amygdala (Right)	67	Precuneus (Left)	3.67	*	*	
46	Cuneus (Right)	63	Supramarginal gyrus (Left)	3.01	*	*	
49	Superior occipital gyrus (Left)	76	Lenticular nucleus, pallidum (Right)	2.72	*		
51	Middle occipital gyrus (Left)	74	Lenticular nucleus, putamen (Right)	2.83	*		
51	Middle occipital gyrus (Left)	80	Heschl gyrus (Right)	2.98	*	*	
51	Middle occipital gyrus (Left)	82	Superior temporal gyrus (Right)	3.15	*	*	
52	Middle occipital gyrus (Right)	80	Heschl gyrus (Right)	3.39	*	*	
57	Postcentral gyrus (Left)	76	Lenticular nucleus, pallidum (Right)	2.90	*		
58	Postcentral gyrus (Right)	63	Supramarginal gyrus (Left)	2.69	*		
58	Postcentral gyrus (Right)	74	Lenticular nucleus, putamen (Right)	2.87	*		
58	Postcentral gyrus (Right)	76	Lenticular nucleus, pallidum (Right)	2.94	*	*	
67	Precuneus (Left)	73	Lenticular nucleus, putamen (Left)	3.46	*	*	
69	Paracentral lobule (Left)	74	Lenticular nucleus, putamen (Right)	3.06	*		

Table S4. Brain Regions Of Interest (ROIs) in functional network components associated with social network embeddedness (low=0, high=1) detected by Network Based Statistics (NBS) (regions from the Anatomical Automatic Labeling atlas)

Brain ROIs				T	Component threshold		
Source		Target			p<0.01	p<0.005	p<0.001
3	Superior frontal gyrus, dorsolateral (Left)	37	Hippocampus (Left)	2.76	*		
3	Superior frontal gyrus, dorsolateral (Left)	40	Parahippocampal gyrus (Right)	2.83	*		
8	Middle frontal gyrus (Right)	58	Postcentral gyrus (Right)	3.14	*	*	
12	Inferior frontal gyrus, opercular part (Right)	33	Median cingulate and paracingulate gyri (Left)	3.53	*	*	
12	Inferior frontal gyrus, opercular part (Right)	40	Parahippocampal gyrus (Right)	2.97	*	*	
12	Inferior frontal gyrus, opercular part (Right)	58	Postcentral gyrus (Right)	2.85	*		
12	Inferior frontal gyrus, opercular part (Right)	77	Thalamus (Left)	2.78	*		
12	Inferior frontal gyrus, opercular part (Right)	78	Thalamus (Right)	3.19	*	*	
14	Inferior frontal gyrus, triangular part (Right)	33	Median cingulate and paracingulate gyri (Left)	3.47	*	*	
14	Inferior frontal gyrus, triangular part (Right)	58	Postcentral gyrus (Right)	2.90	*		
14	Inferior frontal gyrus, triangular part (Right)	63	Supramarginal gyrus (Left)	3.43	*	*	
14	Inferior frontal gyrus, triangular part (Right)	64	Supramarginal gyrus (Right)	3.01	*	*	
14	Inferior frontal gyrus, triangular part (Right)	77	Thalamus (Left)	2.99	*	*	
14	Inferior frontal gyrus, triangular part (Right)	78	Thalamus (Right)	2.82	*		
16	Inferior frontal gyrus, orbital part (Right)	38	Hippocampus (Right)	2.83	*		
16	Inferior frontal gyrus, orbital part (Right)	58	Postcentral gyrus (Right)	3.10	*	*	
17	Rolandic operculum (Left)	77	Thalamus (Left)	3.17	*	*	
18	Rolandic operculum (Right)	77	Thalamus (Left)	3.22	*	*	
22	Olfactory cortex (Right)	69	Paracentral lobule (Left)	2.74	*		
22	Olfactory cortex (Right)	70	Paracentral lobule (Right)	2.69	*		
29	Insula (Left)	45	Cuneus (Left)	3.37	*	*	
29	Insula (Left)	46	Cuneus (Right)	3.29	*	*	
29	Insula (Left)	50	Superior occipital gyrus (Right)	3.10	*	*	
29	Insula (Left)	52	Middle occipital gyrus (Right)	3.09	*	*	
29	Insula (Left)	60	Superior parietal gyrus (Right)	2.74	*		

29	Insula (Left)	67	Precuneus (Left)	3.91	*	*	*
29	Insula (Left)	68	Precuneus (Right)	3.48	*	*	*
30	Insula (Right)	45	Cuneus (Left)	3.17	*	*	
30	Insula (Right)	46	Cuneus (Right)	2.90	*		
30	Insula (Right)	50	Superior occipital gyrus (Right)	2.81	*		
30	Insula (Right)	52	Middle occipital gyrus (Right)	2.88	*		
30	Insula (Right)	67	Precuneus (Left)	3.02	*	*	
30	Insula (Right)	68	Precuneus (Right)	2.70	*		
31	Anterior cingulate and paracingulate gyri (Left)	59	Superior parietal gyrus (Left)	3.73	*	*	*
31	Anterior cingulate and paracingulate gyri (Left)	60	Superior parietal gyrus (Right)	3.12	*	*	
31	Anterior cingulate and paracingulate gyri (Left)	66	Angular gyrus (Right)	3.61	*	*	*
31	Anterior cingulate and paracingulate gyri (Left)	67	Precuneus (Left)	3.97	*	*	*
31	Anterior cingulate and paracingulate gyri (Left)	68	Precuneus (Right)	3.24	*	*	
32	Anterior cingulate and paracingulate gyri (Right)	58	Postcentral gyrus (Right)	3.11	*	*	
32	Anterior cingulate and paracingulate gyri (Right)	59	Superior parietal gyrus (Left)	3.87	*	*	*
32	Anterior cingulate and paracingulate gyri (Right)	60	Superior parietal gyrus (Right)	3.18	*	*	
32	Anterior cingulate and paracingulate gyri (Right)	66	Angular gyrus (Right)	3.03	*	*	
32	Anterior cingulate and paracingulate gyri (Right)	67	Precuneus (Left)	3.99	*	*	*
32	Anterior cingulate and paracingulate gyri (Right)	68	Precuneus (Right)	2.85	*		
32	Anterior cingulate and paracingulate gyri (Right)	70	Paracentral lobule (Right)	2.75	*		
33	Median cingulate and paracingulate gyri (Left)	60	Superior parietal gyrus (Right)	2.97	*	*	
33	Median cingulate and paracingulate gyri (Left)	71	Caudate nucleus (Left)	2.81	*		
34	Median cingulate and paracingulate gyri (Right)	58	Postcentral gyrus (Right)	2.72	*		
34	Median cingulate and paracingulate gyri (Right)	59	Superior parietal gyrus (Left)	3.07	*	*	
34	Median cingulate and paracingulate gyri (Right)	60	Superior parietal gyrus (Right)	4.53	*	*	
34	Median cingulate and paracingulate gyri (Right)	64	Supramarginal gyrus (Right)	4.05	*	*	
34	Median cingulate and paracingulate gyri (Right)	67	Precuneus (Left)	2.80	*		
34	Median cingulate and paracingulate gyri (Right)	68	Precuneus (Right)	2.84	*		
41	Amygdala (Left)	45	Cuneus (Left)	3.16	*	*	
42	Amygdala (Right)	67	Precuneus (Left)	3.30	*	*	

45	Cuneus (Left)	63	Supramarginal gyrus (Left)	2.89	*		
57	Postcentral gyrus (Left)	73	Lenticular nucleus, putamen (Left)	2.72	*		
57	Postcentral gyrus (Left)	76	Lenticular nucleus, pallidum (Right)	2.98	*	*	
58	Postcentral gyrus (Right)	71	Caudate nucleus (Left)	2.80	*		
58	Postcentral gyrus (Right)	76	Lenticular nucleus, pallidum (Right)	3.33	*	*	
59	Superior parietal gyrus (Left)	71	Caudate nucleus (Left)	3.83	*	*	*
59	Superior parietal gyrus (Left)	72	Caudate nucleus (Right)	3.44	*	*	
59	Superior parietal gyrus (Left)	73	Lenticular nucleus, putamen (Left)	2.86	*		
60	Superior parietal gyrus (Right)	71	Caudate nucleus (Left)	3.02	*	*	
60	Superior parietal gyrus (Right)	72	Caudate nucleus (Right)	2.85	*		
67	Precuneus (Left)	71	Caudate nucleus (Left)	3.97	*	*	*
67	Precuneus (Left)	72	Caudate nucleus (Right)	3.76	*	*	*
67	Precuneus (Left)	88	Temporal pole: middle temporal gyrus (Right)	2.98	*	*	
68	Precuneus (Right)	71	Caudate nucleus (Left)	3.58	*	*	*
68	Precuneus (Right)	72	Caudate nucleus (Right)	3.72	*	*	*
69	Paracentral lobule (Left)	71	Caudate nucleus (Left)	2.95	*	*	
69	Paracentral lobule (Left)	72	Caudate nucleus (Right)	2.83	*		
76	Lenticular nucleus, pallidum (Right)	82	Superior temporal gyrus (Right)	2.97	*	*	

Table S5. OLS regression analyses predicting mean functional connectivity of components associated with individual characteristics detected by Network Based Statistics (NBS) (n=64) (regions from the Anatomical Automatic Labeling atlas)

	(1) Age (p<0.01)	(2) Age (p<0.005)	(3) Age (p<0.001)	(4) Social network size (p<0.001)	(5) Social network embeddedness (continuous) (p<0.01)	(6) Social network embeddedness (continuous) (p<0.005)	(7) Social network embeddedness (continuous) (p<0.001)	(8) Social network embeddedness (low=0, high=1) (p<0.01)	(9) Social network embeddedness (low=0, high=1) (p<0.005)	(10) Social network embeddedness (low=0, high=1) (p<0.001)
Female	0.014	0.027	0.045	0.093	0.020	0.015	0.009	0.057	0.060	0.085
	(0.66)	(1.13)	(0.94)	(2.39)	(1.11)	(0.70)	(0.31)	(2.78)	(2.74)	(2.73)
MMSE score	-0.005	-0.005	0.004	-0.009	0.005	0.007	0.006	0.002	0.002	0.005
	(-1.44)	(-1.08)	(0.41)	(-1.31)	(1.67)	(1.90)	(1.18)	(0.56)	(0.50)	(0.88)
Age	-0.012*	-0.012*	-0.016*	-0.010*	-0.002	-0.002	-0.004	0.002	0.001	0.000
	(-8.57)	(-7.54)	(-4.99)	(-3.59)	(-1.45)	(-1.17)	(-1.77)	(1.20)	(0.72)	(0.17)
Years of education	0.003	0.001	0.010	0.008	0.001	-0.000	-0.001	0.004	0.003	0.002
	(0.85)	(0.20)	(1.38)	(1.34)	(0.25)	(-0.13)	(-0.27)	(1.24)	(0.92)	(0.36)
Social network size	0.004	0.004	0.002	0.030*	-0.018*	-0.019*	-0.021*	-0.012*	-0.013*	-0.014*
	(1.22)	(1.29)	(0.35)	(5.25)	(-6.91)	(-6.08)	(-4.86)	(-4.95)	(-4.97)	(-3.65)
Social network embeddedness (continuous)	-0.006	-0.007	-0.005	-0.056*	0.057*	0.059*	0.064*			
	(-0.82)	(-0.86)	(-0.29)	(-4.13)	(9.03)	(7.96)	(6.20)			
Social network embeddedness (low=0, high=1)								0.223*	0.233*	0.259*
								(8.37)	(8.20)	(6.41)
Intercept	1.048*	0.973*	1.153	0.861	-0.129	-0.189	-0.063	-0.269	-0.233	-0.396
	(6.69)	(5.41)	(3.15)	(2.91)	(-0.94)	(-1.17)	(-0.28)	(-1.77)	(-1.43)	(-1.71)
R^2	0.592	0.534	0.406	0.418	0.634	0.573	0.466	0.595	0.590	0.504

Note. T statistics in parentheses; * p<0.001

Table S6. OLS regression analyses predicting mean functional connectivity of components associated with individual characteristics detected by Network Based Statistics (NBS) after excluding outliers (|DFBETA|>0.25) (regions from the Anatomical Automatic Labeling atlas)

	(1) Age (p<0.01)	(2) Age (p<0.005)	(3) Age (p<0.001)	(4) Social network size (p<0.001)	(5) Social network embeddedness (continuous) (p<0.01)	(6) Social network embeddedness (continuous) (p<0.005)	(7) Social network embeddedness (continuous) (p<0.001)
Female	0.018	0.033	0.093	0.077	0.016	0.007	0.005
	(0.90)	(1.42)	(2.09)	(2.05)	(0.88)	(0.32)	(0.18)
MMSE score	-0.006	-0.004	0.004	-0.005	0.006	0.007	0.005
	(-1.55)	(-1.01)	(0.45)	(-0.79)	(1.80)	(1.92)	(0.88)
Age	-0.012*	-0.012*	-0.018*	-0.011*	-0.002	-0.002	-0.005
	(-8.35)	(-7.18)	(-5.49)	(-4.33)	(-1.23)	(-1.24)	(-2.30)
Years of	0.002	0.002	0.009	0.003	-0.000	-0.001	-0.002
education	(0.76)	(0.50)	(1.34)	(0.59)	(-0.07)	(-0.36)	(-0.44)
Social network	0.003	0.005	0.001	0.028*	-0.016*	-0.014	-0.016
size	(0.87)	(1.42)	(0.14)	(3.70)	(-3.75)	(-2.73)	(-2.22)
Social network	-0.003	-0.006	0.002	-0.054	0.052*	0.049*	0.054*
embeddedness (continuous)	(-0.37)	(-0.76)	(0.16)	(-3.46)	(5.91)	(4.75)	(3.65)
Intercept	1.048*	0.915*	1.214	0.906	-0.139	-0.161	0.091
	(6.53)	(5.06)	(3.42)	(3.23)	(-1.03)	(-1.00)	(0.39)
N	61	61	59	61	58	60	58
R^2	0.602	0.541	0.512	0.412	0.561	0.496	0.410

Note. T statistics in parentheses; * p<0.001

Table S7. Network Based Statistics (NBS) for testing component-level interactions between age and other individual characteristics (n=64) (regions from the Anatomical Automatic Labeling atlas)

Variables	Association	Statistics	Component threshold p<0.01		p<0.005		p<0.001	
			Size	(p_{NBS})	Size	(p_{NBS})	Size	(p_{NBS})
Years of education	+	Extent	18	(0.240)	5	(0.384)	2	(0.270)
		Intensity	5.2	(0.306)	1.3	(0.447)	0.2	(0.441)
Social network size	+	Extent	3	(0.809)	1	(0.946)	1	(0.622)
		Intensity	1.0	(0.760)	0.7	(0.653)	0.1	(0.517)
Social network embeddedness (continuous)	+	Extent	2	(0.921)	1	(0.954)	0	(1.000)
		Intensity	0.5	(0.902)	0.2	(0.867)	0.0	(1.000)
Social network embeddedness (low=0, high=1)	+	Extent	8	(0.485)	3	(0.580)	0	(1.000)
		Intensity	2.1	(0.554)	0.6	(0.680)	0.0	(1.000)

Table S8. Network Based Statistics (NBS) for testing component-level associations between functional connectivity and individual characteristics (n=64) (regions from the Harvard-Oxford probabilistic atlas)

Variables	Association	Statistics	Component threshold					
			p<0.01		p<0.005		p<0.001	
			Size	(p_{NBS})	Size	(p_{NBS})	Size	(p_{NBS})
Age	-	Extent	114	(0.007)	59	(0.010)	13	(0.008)
		Intensity	47.4	(0.010)	24.7	(0.009)	6.2	(0.006)
Years of education	+	Extent	75	(0.037)	43	(0.024)	9	(0.023)
		Intensity	35.8	(0.023)	20.4	(0.018)	6.6	(0.006)
Social network size	+	Extent	15	(0.382)	3	(0.662)	0	(1.000)
		Intensity	3.8	(0.481)	0.9	(0.669)	0.0	(1.000)
Social network embeddedness (continuous)	+	Extent	58	(0.072)	25	(0.071)	3	(0.176)
		Intensity	20.5	(0.089)	8.4	(0.089)	1.0	(0.231)
Social network embeddedness (low=0, high=1)	+	Extent	68	(0.042)	41	(0.026)	4	(0.094)
		Intensity	26.8	(0.045)	12.9	(0.042)	2.2	(0.049)

Table S9. Network Based Statistics (NBS) for testing component-level interactions between age and other individual characteristics (n=64) (regions from the Harvard-Oxford probabilistic atlas)

Variables	Association	Statistics	Component threshold					
			p<0.01		p<0.005		p<0.001	
			Size	(p_{NBS})	Size	(p_{NBS})	Size	(p_{NBS})
Years of education	+	Extent	12	(0.465)	4	(0.559)	0	(1.000)
		Intensity	3.4	(0.525)	0.9	(0.661)	0.0	(1.000)
Social network size	+	Extent	9	(0.570)	4	(0.572)	0	(1.000)
		Intensity	2.5	(0.622)	0.7	(0.725)	0.0	(1.000)
Social network embeddedness (continuous)	+	Extent	22	(0.275)	3	(0.650)	1	(0.711)
		Intensity	5.5	(0.372)	1.2	(0.533)	0.2	(0.524)
Social network embeddedness (low=0, high=1)	+	Extent	8	(0.577)	1	(0.972)	1	(0.703)
		Intensity	2.8	(0.564)	1.3	(0.536)	0.7	(0.253)

Table S10. Network Based Statistics (NBS) for testing component-level associations between functional connectivity and individual characteristics (n=64) (regions from the Dosenbach atlas)

Variables	Association	Statistics	Component threshold p<0.01 Size	(p_{NBS})	p<0.005 Size	(p_{NBS})	p<0.001 Size	(p_{NBS})
Age	-	Extent	117	(0.054)	59	(0.052)	6	(0.112)
		Intensity	43.6	(0.062)	20.1	(0.064)	1.2	(0.234)
Years of education	+	Extent	74	(0.155)	34	(0.137)	5	(0.156)
		Intensity	24.8	(0.181)	11.4	(0.151)	0.3	(0.608)
Social network size	+	Extent	9	(0.771)	4	(0.767)	1	(0.866)
		Intensity	2.8	(0.776)	1.5	(0.688)	0.4	(0.568)
Social network embeddedness (continuous)	+	Extent	67	(0.187)	23	(0.190)	2	(0.499)
		Intensity	24.8	(0.179)	8.2	(0.201)	1.1	(0.254)
Social network embeddedness (low=0, high=1)	+	Extent	62	(0.203)	12	(0.366)	4	(0.208)
		Intensity	25.8	(0.163)	4.6	(0.340)	1.1	(0.265)

Table S11. Network Based Statistics (NBS) for testing component-level interactions between age and other individual characteristics (n=64) (regions from the Dosenbach atlas)

Variables	Association	Statistics	Component threshold p<0.01 Size	(p_{NBS})	p<0.005 Size	(p_{NBS})	p<0.001 Size	(p_{NBS})
Years of education	+	Extent	18	(0.564)	7	(0.542)	1	(0.850)
		Intensity	6.4	(0.568)	1.9	(0.601)	0.4	(0.557)
Social network size	+	Extent	10	(0.730)	4	(0.756)	2	(0.494)
		Intensity	3.2	(0.715)	2.2	(0.560)	0.9	(0.294)
Social network embeddedness (continuous)	+	Extent	26	(0.475)	13	(0.337)	2	(0.497)
		Intensity	9.9	(0.426)	4.7	(0.332)	0.6	(0.433)
Social network embeddedness (low=0, high=1)	+	Extent	10	(0.728)	8	(0.507)	3	(0.314)
		Intensity	6.1	(0.562)	3.9	(0.383)	1.2	(0.230)

Table S12. Brain Regions Of Interest (ROIs) in functional network components associated with age detected by Network Based Statistics (NBS) (regions from the Harvard-Oxford probabilistic atlas)

Brain ROIs				T	Component threshold		
	Source		Target		p<0.01	p<0.005	p<0.001
1	Frontal Pole (Right)	40	Supramarginal Gyrus, posterior division (Left)	-2.83	*		
2	Frontal Pole (Left)	88	Supracalcarine Cortex (Right)	-2.68	*		
2	Frontal Pole (Left)	94	Caudate (Right)	-2.73	*		
3	Insular Cortex (Right)	77	Frontal Operculum Cortex (Left)	-3.04	*	*	
4	Insular Cortex (Left)	37	Supramarginal Gyrus, anterior division (Right)	-2.74	*		
4	Insular Cortex (Left)	39	Supramarginal Gyrus, posterior division (Right)	-2.70	*		
4	Insular Cortex (Left)	41	Angular Gyrus (Right)	-2.67	*		
4	Insular Cortex (Left)	51	Juxtapositional Lobule Cortex (Left)	-2.74	*		
4	Insular Cortex (Left)	56	Cingulate Gyrus, posterior division	-2.89	*		
5	Superior Frontal Gyrus (Right)	12	Inferior Frontal Gyrus, pars opercularis (Left)	-2.76	*		
6	Superior Frontal Gyrus (Left)	34	Postcentral Gyrus (Left)	-3.03	*	*	
6	Superior Frontal Gyrus (Left)	37	Supramarginal Gyrus, anterior division (Right)	-2.78	*		
6	Superior Frontal Gyrus (Left)	38	Supramarginal Gyrus, anterior division (Left)	-2.84	*		
6	Superior Frontal Gyrus (Left)	47	Intracalcarine Cortex (Right)	-2.69	*		
6	Superior Frontal Gyrus (Left)	48	Intracalcarine Cortex (Left)	-2.81	*		
6	Superior Frontal Gyrus (Left)	80	Parietal Operculum Cortex (Right)	-2.82	*		
6	Superior Frontal Gyrus (Left)	81	Parietal Operculum Cortex (Left)	-2.92	*	*	
6	Superior Frontal Gyrus (Left)	84	Heschl's Gyrus (Right)	-2.68	*		
6	Superior Frontal Gyrus (Left)	88	Supracalcarine Cortex (Right)	-2.69	*		
7	Middle Frontal Gyrus (Right)	46	Lateral Occipital Cortex, inferior division (Left)	-2.99	*		
7	Middle Frontal Gyrus (Right)	91	Occipital Pole (Left)	-3.30	*		
8	Middle Frontal Gyrus (Left)	23	Middle Temporal Gyrus, posterior division (Right)	-3.00	*	*	
8	Middle Frontal Gyrus (Left)	25	Middle Temporal Gyrus, temporooccipital part (Right)	-2.79	*		
8	Middle Frontal Gyrus (Left)	29	Inferior Temporal Gyrus, posterior division (Right)	-2.75	*		

8	Middle Frontal Gyrus (Left)	41	Angular Gyrus (Right)	-2.78	*		
8	Middle Frontal Gyrus (Left)	59	Cuneal Cortex (Left)	-2.78	*		
8	Middle Frontal Gyrus (Left)	60	Frontal Orbital Cortex (Right)	-2.81	*		
8	Middle Frontal Gyrus (Left)	81	Parietal Operculum Cortex (Left)	-2.73	*		
8	Middle Frontal Gyrus (Left)	82	Planum Polare (Right)	-3.28	*	*	
10	Inferior Frontal Gyrus, pars triangularis (Left)	82	Planum Polare (Right)	-3.07	*	*	
11	Inferior Frontal Gyrus, pars opercularis (Right)	24	Middle Temporal Gyrus, posterior division (Left)	-2.72	*		
12	Inferior Frontal Gyrus, pars opercularis (Left)	39	Supramarginal Gyrus, posterior division (Right)	-2.83	*		
12	Inferior Frontal Gyrus, pars opercularis (Left)	41	Angular Gyrus (Right)	-2.94	*	*	
12	Inferior Frontal Gyrus, pars opercularis (Left)	82	Planum Polare (Right)	-3.38	*	*	
12	Inferior Frontal Gyrus, pars opercularis (Left)	84	Heschl's Gyrus (Right)	-2.97	*	*	
12	Inferior Frontal Gyrus, pars opercularis (Left)	89	Supracalcarine Cortex (Left)	-2.95	*	*	
13	Precentral Gyrus (Right)	29	Inferior Temporal Gyrus, posterior division (Right)	-4.72	*	*	*
14	Precentral Gyrus (Left)	23	Middle Temporal Gyrus, posterior division (Right)	-2.71	*		
14	Precentral Gyrus (Left)	29	Inferior Temporal Gyrus, posterior division (Right)	-4.92	*	*	*
14	Precentral Gyrus (Left)	62	Parahippocampal Gyrus, anterior division (Right)	-2.85	*		
14	Precentral Gyrus (Left)	63	Parahippocampal Gyrus, anterior division (Left)	-2.90	*		
14	Precentral Gyrus (Left)	81	Parietal Operculum Cortex (Left)	-2.79	*		
16	Temporal Pole (Left)	71	Temporal Fusiform Cortex, posterior division (Left)	-2.76	*		
19	Superior Temporal Gyrus, posterior division (Right)	51	Juxtapositional Lobule Cortex (Left)	-2.97	*	*	
23	Middle Temporal Gyrus, posterior division (Right)	51	Juxtapositional Lobule Cortex (Left)	-3.55	*	*	*
24	Middle Temporal Gyrus, posterior division (Left)	84	Heschl's Gyrus (Right)	-2.69	*		
25	Middle Temporal Gyrus, temporooccipital part (Right)	53	Paracingulate Gyrus (Right)	-3.57	*	*	
25	Middle Temporal Gyrus, temporooccipital part (Right)	54	Paracingulate Gyrus (Left)	-3.46	*	*	

26	Middle Temporal Gyrus, temporooccipital part (Left)	53	Paracingulate Gyrus (Right)	-3.50	*	*	
26	Middle Temporal Gyrus, temporooccipital part (Left)	96	Putamen (Right)	-3.12	*	*	
29	Inferior Temporal Gyrus, posterior division (Right)	33	Postcentral Gyrus (Right)	-3.45	*	*	
29	Inferior Temporal Gyrus, posterior division (Right)	34	Postcentral Gyrus (Left)	-4.40	*	*	*
29	Inferior Temporal Gyrus, posterior division (Right)	36	Superior Parietal Lobule (Left)	-2.82	*		
29	Inferior Temporal Gyrus, posterior division (Right)	50	Juxtapositional Lobule Cortex (Right)	-3.87	*	*	*
29	Inferior Temporal Gyrus, posterior division (Right)	51	Juxtapositional Lobule Cortex (Left)	-3.90	*	*	*
29	Inferior Temporal Gyrus, posterior division (Right)	79	Central Opercular Cortex (Left)	-2.97	*	*	
30	Inferior Temporal Gyrus, posterior division (Left)	37	Supramarginal Gyrus, anterior division (Right)	-2.90	*		
30	Inferior Temporal Gyrus, posterior division (Left)	50	Juxtapositional Lobule Cortex (Right)	-2.98	*	*	
34	Postcentral Gyrus (Left)	63	Parahippocampal Gyrus, anterior division (Left)	-2.82	*		
36	Superior Parietal Lobule (Left)	63	Parahippocampal Gyrus, anterior division (Left)	-3.53	*	*	
36	Superior Parietal Lobule (Left)	78	Central Opercular Cortex (Right)	-2.75	*		
37	Supramarginal Gyrus, anterior division (Right)	62	Parahippocampal Gyrus, anterior division (Right)	-2.83	*		
37	Supramarginal Gyrus, anterior division (Right)	77	Frontal Operculum Cortex (Left)	-3.93	*	*	*
37	Supramarginal Gyrus, anterior division (Right)	78	Central Opercular Cortex (Right)	-3.58	*	*	*
37	Supramarginal Gyrus, anterior division (Right)	79	Central Opercular Cortex (Left)	-3.11	*	*	
37	Supramarginal Gyrus, anterior division (Right)	83	Planum Polare (Left)	-3.91	*	*	*
38	Supramarginal Gyrus, anterior division (Left)	77	Frontal Operculum Cortex (Left)	-2.91	*		
39	Supramarginal Gyrus, posterior division (Right)	77	Frontal Operculum Cortex (Left)	-3.15	*	*	
40	Supramarginal Gyrus, posterior division (Left)	53	Paracingulate Gyrus (Right)	-3.28	*	*	
40	Supramarginal Gyrus, posterior division (Left)	55	Cingulate Gyrus, anterior division	-2.84	*		
41	Angular Gyrus (Right)	76	Frontal Operculum Cortex (Right)	-2.94	*	*	
42	Angular Gyrus (Left)	53	Paracingulate Gyrus (Right)	-3.50	*	*	
42	Angular Gyrus (Left)	55	Cingulate Gyrus, anterior division	-2.95	*	*	

42	Angular Gyrus (Left)	94	Caudate (Right)	-2.84	*		
42	Angular Gyrus (Left)	96	Putamen (Right)	-3.42	*	*	
42	Angular Gyrus (Left)	99	Pallidum (Left)	-3.04	*	*	
48	Intracalcarine Cortex (Left)	54	Paracingulate Gyrus (Left)	-3.06	*	*	
48	Intracalcarine Cortex (Left)	63	Parahippocampal Gyrus, anterior division (Left)	-2.78	*		
50	Juxtapositional Lobule Cortex (Right)	62	Parahippocampal Gyrus, anterior division (Right)	-2.96	*	*	
50	Juxtapositional Lobule Cortex (Right)	63	Parahippocampal Gyrus, anterior division (Left)	-3.05	*	*	
50	Juxtapositional Lobule Cortex (Right)	82	Planum Polare (Right)	-2.90	*		
50	Juxtapositional Lobule Cortex (Right)	83	Planum Polare (Left)	-3.56	*	*	*
50	Juxtapositional Lobule Cortex (Right)	87	Planum Temporale (Left)	-3.15	*	*	
51	Juxtapositional Lobule Cortex (Left)	62	Parahippocampal Gyrus, anterior division (Right)	-3.48	*	*	*
51	Juxtapositional Lobule Cortex (Left)	63	Parahippocampal Gyrus, anterior division (Left)	-3.47	*	*	
51	Juxtapositional Lobule Cortex (Left)	67	Lingual Gyrus (Left)	-2.84	*		
51	Juxtapositional Lobule Cortex (Left)	70	Temporal Fusiform Cortex, posterior division (Right)	-2.91	*		
51	Juxtapositional Lobule Cortex (Left)	82	Planum Polare (Right)	-3.76	*	*	*
51	Juxtapositional Lobule Cortex (Left)	83	Planum Polare (Left)	-3.05	*	*	
51	Juxtapositional Lobule Cortex (Left)	84	Heschl's Gyrus (Right)	-3.43	*	*	
51	Juxtapositional Lobule Cortex (Left)	85	Heschl's Gyrus (Left)	-3.72	*	*	*
51	Juxtapositional Lobule Cortex (Left)	87	Planum Temporale (Left)	-2.84	*		
53	Paracingulate Gyrus (Right)	91	Occipital Pole (Left)	-2.75	*		
54	Paracingulate Gyrus (Left)	90	Occipital Pole (Right)	-2.81	*		
54	Paracingulate Gyrus (Left)	100	Hippocampus (Right)	-2.94	*	*	
55	Cingulate Gyrus, anterior division	56	Cingulate Gyrus, posterior division	-2.77	*		
55	Cingulate Gyrus, anterior division	61	Frontal Orbital Cortex (Left)	-2.82	*		
55	Cingulate Gyrus, anterior division	63	Parahippocampal Gyrus, anterior division (Left)	-2.84	*		
55	Cingulate Gyrus, anterior division	77	Frontal Operculum Cortex (Left)	-2.83	*		
56	Cingulate Gyrus, posterior division	76	Frontal Operculum Cortex (Right)	-3.40	*	*	
56	Cingulate Gyrus, posterior division	96	Putamen (Right)	-3.17	*	*	
58	Cuneal Cortex (Right)	63	Parahippocampal Gyrus, anterior division (Left)	-3.01	*	*	

58	Cuneal Cortex (Right)	82	Planum Polare (Right)	-3.18	*	*
59	Cuneal Cortex (Left)	63	Parahippocampal Gyrus, anterior division (Left)	-3.45	*	*
61	Frontal Orbital Cortex (Left)	82	Planum Polare (Right)	-3.27	*	*
61	Frontal Orbital Cortex (Left)	94	Caudate (Right)	-3.26	*	*
63	Parahippocampal Gyrus, anterior division (Left)	96	Putamen (Right)	-2.84	*	
63	Parahippocampal Gyrus, anterior division (Left)	98	Pallidum (Right)	-3.30	*	*
63	Parahippocampal Gyrus, anterior division (Left)	99	Pallidum (Left)	-2.86	*	
71	Temporal Fusiform Cortex, posterior division (Left)	98	Pallidum (Right)	-2.95	*	*
77	Frontal Operculum Cortex (Left)	82	Planum Polare (Right)	-3.07	*	*
77	Frontal Operculum Cortex (Left)	84	Heschl's Gyrus (Right)	-3.04	*	*
77	Frontal Operculum Cortex (Left)	89	Supracalcarine Cortex (Left)	-2.67	*	
79	Central Opercular Cortex (Left)	84	Heschl's Gyrus (Right)	-2.81	*	

Table S13. Brain Regions Of Interest (ROIs) in functional network components associated with years of education detected by Network Based Statistics (NBS) (regions from the Harvard-Oxford probabilistic atlas)

Brain ROIs				T	Component threshold		
Source		Target			p<0.01	p<0.005	p<0.001
3	Insular Cortex (Right)	80	Parietal Operculum Cortex (Right)	3.15	*	*	
6	Superior Frontal Gyrus (Left)	23	Middle Temporal Gyrus, posterior division (Right)	2.95	*		
6	Superior Frontal Gyrus (Left)	29	Inferior Temporal Gyrus, posterior division (Right)	3.83	*		
6	Superior Frontal Gyrus (Left)	32	Inferior Temporal Gyrus, temporooccipital part (Left)	2.71	*		
13	Precentral Gyrus (Right)	14	Precentral Gyrus (Left)	3.20	*	*	
13	Precentral Gyrus (Right)	33	Postcentral Gyrus (Right)	3.28	*	*	
13	Precentral Gyrus (Right)	80	Parietal Operculum Cortex (Right)	3.29	*	*	
13	Precentral Gyrus (Right)	85	Heschl's Gyrus (Left)	2.74	*		
14	Precentral Gyrus (Left)	19	Superior Temporal Gyrus, posterior division (Right)	3.06	*	*	
14	Precentral Gyrus (Left)	32	Inferior Temporal Gyrus, temporooccipital part (Left)	3.12	*	*	
14	Precentral Gyrus (Left)	33	Postcentral Gyrus (Right)	2.77	*		
14	Precentral Gyrus (Left)	35	Superior Parietal Lobule (Right)	2.76	*		
14	Precentral Gyrus (Left)	78	Central Opercular Cortex (Right)	2.80	*		
14	Precentral Gyrus (Left)	80	Parietal Operculum Cortex (Right)	2.96	*	*	
14	Precentral Gyrus (Left)	86	Planum Temporale (Right)	2.84	*		
17	Superior Temporal Gyrus, anterior division (Right)	46	Lateral Occipital Cortex, inferior division (Left)	2.75	*		
17	Superior Temporal Gyrus, anterior division (Right)	81	Parietal Operculum Cortex (Left)	2.82	*		
17	Superior Temporal Gyrus, anterior division (Right)	88	Supracalcarine Cortex (Right)	3.07	*	*	
18	Superior Temporal Gyrus, anterior division (Left)	63	Parahippocampal Gyrus, anterior division (Left)	2.77	*		
18	Superior Temporal Gyrus, anterior division (Left)	66	Lingual Gyrus (Right)	3.00	*	*	
18	Superior Temporal Gyrus, anterior division (Left)	67	Lingual Gyrus (Left)	3.72	*	*	*

18	Superior Temporal Gyrus, anterior division (Left)	101	Hippocampus (Left)	2.74	*		
19	Superior Temporal Gyrus, posterior division (Right)	26	Middle Temporal Gyrus, temporooccipital part (Left)	3.21	*	*	
19	Superior Temporal Gyrus, posterior division (Right)	32	Inferior Temporal Gyrus, temporooccipital part (Left)	3.05	*	*	
19	Superior Temporal Gyrus, posterior division (Right)	46	Lateral Occipital Cortex, inferior division (Left)	3.35	*	*	
19	Superior Temporal Gyrus, posterior division (Right)	47	Intracalcarine Cortex (Right)	3.84	*	*	*
19	Superior Temporal Gyrus, posterior division (Right)	48	Intracalcarine Cortex (Left)	5.26	*	*	*
19	Superior Temporal Gyrus, posterior division (Right)	50	Juxtapositional Lobule Cortex (Right)	2.72	*		
19	Superior Temporal Gyrus, posterior division (Right)	58	Cuneal Cortex (Right)	3.83	*	*	*
19	Superior Temporal Gyrus, posterior division (Right)	59	Cuneal Cortex (Left)	4.26	*	*	*
19	Superior Temporal Gyrus, posterior division (Right)	61	Frontal Orbital Cortex (Left)	3.26	*	*	
19	Superior Temporal Gyrus, posterior division (Right)	66	Lingual Gyrus (Right)	2.75	*		
19	Superior Temporal Gyrus, posterior division (Right)	67	Lingual Gyrus (Left)	4.72	*	*	*
19	Superior Temporal Gyrus, posterior division (Right)	73	Temporal Occipital Fusiform Cortex (Left)	3.50	*	*	*
19	Superior Temporal Gyrus, posterior division (Right)	74	Occipital Fusiform Gyrus (Right)	3.38	*	*	
19	Superior Temporal Gyrus, posterior division (Right)	79	Central Opercular Cortex (Left)	3.09	*	*	
19	Superior Temporal Gyrus, posterior division (Right)	81	Parietal Operculum Cortex (Left)	2.96	*	*	
19	Superior Temporal Gyrus, posterior division (Right)	87	Planum Temporale (Left)	2.99	*	*	
19	Superior Temporal Gyrus, posterior division (Right)	88	Supracalcarine Cortex (Right)	4.74	*	*	*
19	Superior Temporal Gyrus, posterior division	89	Supracalcarine Cortex (Left)	3.95	*	*	*

	(Right)					
20	Superior Temporal Gyrus, posterior division (Left)	47	Intracalcarine Cortex (Right)	2.67	*	
20	Superior Temporal Gyrus, posterior division (Left)	48	Intracalcarine Cortex (Left)	2.76	*	
20	Superior Temporal Gyrus, posterior division (Left)	63	Parahippocampal Gyrus, anterior division (Left)	3.04	*	*
20	Superior Temporal Gyrus, posterior division (Left)	67	Lingual Gyrus (Left)	3.04	*	*
20	Superior Temporal Gyrus, posterior division (Left)	88	Supracalcarine Cortex (Right)	2.88	*	
25	Middle Temporal Gyrus, temporooccipital part (Right)	28	Inferior Temporal Gyrus, anterior division (Left)	2.73	*	
28	Inferior Temporal Gyrus, anterior division (Left)	44	Lateral Occipital Cortex, superior division (Left)	2.74	*	
28	Inferior Temporal Gyrus, anterior division (Left)	45	Lateral Occipital Cortex, inferior division (Right)	2.84	*	
32	Inferior Temporal Gyrus, temporooccipital part (Left)	35	Superior Parietal Lobule (Right)	2.77	*	
32	Inferior Temporal Gyrus, temporooccipital part (Left)	36	Superior Parietal Lobule (Left)	2.93	*	*
32	Inferior Temporal Gyrus, temporooccipital part (Left)	50	Juxtapositional Lobule Cortex (Right)	3.20	*	*
32	Inferior Temporal Gyrus, temporooccipital part (Left)	51	Juxtapositional Lobule Cortex (Left)	3.37	*	*
33	Postcentral Gyrus (Right)	78	Central Opercular Cortex (Right)	2.71	*	
33	Postcentral Gyrus (Right)	79	Central Opercular Cortex (Left)	2.72	*	
33	Postcentral Gyrus (Right)	85	Heschl's Gyrus (Left)	3.31	*	*
33	Postcentral Gyrus (Right)	86	Planum Temporale (Right)	2.82	*	
35	Superior Parietal Lobule (Right)	85	Heschl's Gyrus (Left)	3.01	*	*
35	Superior Parietal Lobule (Right)	86	Planum Temporale (Right)	2.76	*	
45	Lateral Occipital Cortex, inferior division (Right)	69	Temporal Fusiform Cortex, anterior division (Left)	3.83	*	*
58	Cuneal Cortex (Right)	63	Parahippocampal Gyrus, anterior division (Left)	3.31	*	*
58	Cuneal Cortex (Right)	69	Temporal Fusiform Cortex, anterior division (Left)	3.00	*	*

59	Cuneal Cortex (Left)	63	Parahippocampal Gyrus, anterior division (Left)	3.06	*	*
63	Parahippocampal Gyrus, anterior division (Left)	66	Lingual Gyrus (Right)	3.11	*	*
63	Parahippocampal Gyrus, anterior division (Left)	67	Lingual Gyrus (Left)	3.46	*	*
63	Parahippocampal Gyrus, anterior division (Left)	75	Occipital Fusiform Gyrus (Left)	2.91	*	
63	Parahippocampal Gyrus, anterior division (Left)	99	Pallidum (Left)	2.92	*	*
68	Temporal Fusiform Cortex, anterior division (Right)	88	Supracalcarine Cortex (Right)	2.74	*	
69	Temporal Fusiform Cortex, anterior division (Left)	75	Occipital Fusiform Gyrus (Left)	2.69	*	
69	Temporal Fusiform Cortex, anterior division (Left)	88	Supracalcarine Cortex (Right)	2.74	*	
69	Temporal Fusiform Cortex, anterior division (Left)	91	Occipital Pole (Left)	3.35	*	*
78	Central Opercular Cortex (Right)	80	Parietal Operculum Cortex (Right)	2.71	*	
80	Parietal Operculum Cortex (Right)	82	Planum Polare (Right)	3.76	*	*
80	Parietal Operculum Cortex (Right)	85	Heschl's Gyrus (Left)	3.01	*	*
80	Parietal Operculum Cortex (Right)	86	Planum Temporale (Right)	2.81	*	
99	Pallidum (Left)	100	Hippocampus (Right)	2.81	*	

Table S14. Brain Regions Of Interest (ROIs) in functional network components associated with social network embeddedness (low=0, high=1) detected by Network Based Statistics (NBS) (regions from the Harvard-Oxford probabilistic atlas)

Brain ROIs				T	Component threshold		
Source		Target			p<0.01	p<0.005	p<0.001
3	Insular Cortex (Right)	33	Postcentral Gyrus (Right)	2.77	*		
3	Insular Cortex (Right)	35	Superior Parietal Lobule (Right)	2.81	*		
3	Insular Cortex (Right)	43	Lateral Occipital Cortex, superior division (Right)	2.95	*	*	
3	Insular Cortex (Right)	57	Precuneous Cortex	3.08	*	*	
3	Insular Cortex (Right)	72	Temporal Occipital Fusiform Cortex (Right)	2.81	*		
4	Insular Cortex (Left)	57	Precuneous Cortex	3.45	*	*	
5	Superior Frontal Gyrus (Right)	64	Parahippocampal Gyrus, posterior division (Right)	2.67	*		
5	Superior Frontal Gyrus (Right)	102	Amygdala (Right)	2.90	*		
7	Middle Frontal Gyrus (Right)	33	Postcentral Gyrus (Right)	3.01	*		
7	Middle Frontal Gyrus (Right)	39	Supramarginal Gyrus, posterior division (Right)	2.73	*		
9	Inferior Frontal Gyrus, pars triangularis (Right)	31	Inferior Temporal Gyrus, temporooccipital part (Right)	2.92	*	*	
9	Inferior Frontal Gyrus, pars triangularis (Right)	37	Supramarginal Gyrus, anterior division (Right)	2.84	*		
9	Inferior Frontal Gyrus, pars triangularis (Right)	43	Lateral Occipital Cortex, superior division (Right)	3.94	*	*	*
9	Inferior Frontal Gyrus, pars triangularis (Right)	45	Lateral Occipital Cortex, inferior division (Right)	2.78	*		
9	Inferior Frontal Gyrus, pars triangularis (Right)	46	Lateral Occipital Cortex, inferior division (Left)	3.00	*	*	
9	Inferior Frontal Gyrus, pars triangularis (Right)	57	Precuneous Cortex	2.75	*		
9	Inferior Frontal Gyrus, pars triangularis (Right)	58	Cuneal Cortex (Right)	3.40	*	*	
9	Inferior Frontal Gyrus, pars triangularis (Right)	59	Cuneal Cortex (Left)	2.84	*		
9	Inferior Frontal Gyrus, pars triangularis (Right)	66	Lingual Gyrus (Right)	2.72	*		
9	Inferior Frontal Gyrus, pars triangularis (Right)	72	Temporal Occipital Fusiform Cortex (Right)	3.24	*	*	
9	Inferior Frontal Gyrus, pars triangularis (Right)	73	Temporal Occipital Fusiform Cortex (Left)	2.76	*		
9	Inferior Frontal Gyrus, pars triangularis (Right)	88	Supracalcarine Cortex (Right)	2.83	*		
10	Inferior Frontal Gyrus, pars triangularis (Left)	98	Pallidum (Right)	2.74	*		

11	Inferior Frontal Gyrus, pars opercularis (Right)	31	Inferior Temporal Gyrus, temporooccipital part (Right)	2.95	*	*	
11	Inferior Frontal Gyrus, pars opercularis (Right)	32	Inferior Temporal Gyrus, temporooccipital part (Left)	3.02	*	*	
11	Inferior Frontal Gyrus, pars opercularis (Right)	35	Superior Parietal Lobule (Right)	3.09	*	*	
11	Inferior Frontal Gyrus, pars opercularis (Right)	43	Lateral Occipital Cortex, superior division (Right)	2.95	*	*	
11	Inferior Frontal Gyrus, pars opercularis (Right)	48	Intracalcarine Cortex (Left)	2.92	*	*	
11	Inferior Frontal Gyrus, pars opercularis (Right)	50	Juxtapositional Lobule Cortex (Right)	2.83	*		
11	Inferior Frontal Gyrus, pars opercularis (Right)	64	Parahippocampal Gyrus, posterior division (Right)	2.88	*		
11	Inferior Frontal Gyrus, pars opercularis (Right)	88	Supracalcarine Cortex (Right)	2.84	*		
11	Inferior Frontal Gyrus, pars opercularis (Right)	89	Supracalcarine Cortex (Left)	3.25	*	*	
12	Inferior Frontal Gyrus, pars opercularis (Left)	72	Temporal Occipital Fusiform Cortex (Right)	3.69	*	*	
12	Inferior Frontal Gyrus, pars opercularis (Left)	98	Pallidum (Right)	2.68	*		
31	Inferior Temporal Gyrus, temporooccipital part (Right)	55	Cingulate Gyrus, anterior division	3.03	*	*	
33	Postcentral Gyrus (Right)	55	Cingulate Gyrus, anterior division	2.77	*		
35	Superior Parietal Lobule (Right)	55	Cingulate Gyrus, anterior division	4.11	*	*	*
35	Superior Parietal Lobule (Right)	95	Caudate (Left)	2.93	*	*	
36	Superior Parietal Lobule (Left)	55	Cingulate Gyrus, anterior division	2.87	*		
36	Superior Parietal Lobule (Left)	94	Caudate (Right)	3.32	*	*	
36	Superior Parietal Lobule (Left)	95	Caudate (Left)	3.51	*	*	
43	Lateral Occipital Cortex, superior division (Right)	53	Paracingulate Gyrus (Right)	2.98	*	*	
43	Lateral Occipital Cortex, superior division (Right)	54	Paracingulate Gyrus (Left)	2.72	*		
43	Lateral Occipital Cortex, superior division (Right)	55	Cingulate Gyrus, anterior division	4.35	*	*	*
43	Lateral Occipital Cortex, superior division (Right)	94	Caudate (Right)	2.92	*	*	
43	Lateral Occipital Cortex, superior division (Right)	95	Caudate (Left)	2.73	*		
44	Lateral Occipital Cortex, superior division (Left)	53	Paracingulate Gyrus (Right)	2.79	*		

44	Lateral Occipital Cortex, superior division (Left)	55	Cingulate Gyrus, anterior division	3.19	*	*	
46	Lateral Occipital Cortex, inferior division (Left)	76	Frontal Operculum Cortex (Right)	3.32	*	*	
47	Intracalcarine Cortex (Right)	76	Frontal Operculum Cortex (Right)	2.71	*		
49	Frontal Medial Cortex	95	Caudate (Left)	3.00	*	*	
55	Cingulate Gyrus, anterior division	57	Precuneous Cortex	3.63	*	*	*
55	Cingulate Gyrus, anterior division	72	Temporal Occipital Fusiform Cortex (Right)	3.12	*	*	
57	Precuneous Cortex	76	Frontal Operculum Cortex (Right)	3.19	*	*	
57	Precuneous Cortex	94	Caudate (Right)	3.44	*	*	
57	Precuneous Cortex	95	Caudate (Left)	2.87	*		
58	Cuneal Cortex (Right)	76	Frontal Operculum Cortex (Right)	3.30	*	*	
59	Cuneal Cortex (Left)	76	Frontal Operculum Cortex (Right)	3.57	*	*	
60	Frontal Orbital Cortex (Right)	68	Temporal Fusiform Cortex, anterior division (Right)	2.70	*		
60	Frontal Orbital Cortex (Right)	78	Central Opercular Cortex (Right)	2.95	*	*	
66	Lingual Gyrus (Right)	76	Frontal Operculum Cortex (Right)	3.15	*	*	
67	Lingual Gyrus (Left)	76	Frontal Operculum Cortex (Right)	2.96	*	*	
72	Temporal Occipital Fusiform Cortex (Right)	76	Frontal Operculum Cortex (Right)	3.05	*	*	
72	Temporal Occipital Fusiform Cortex (Right)	78	Central Opercular Cortex (Right)	2.99	*	*	
72	Temporal Occipital Fusiform Cortex (Right)	79	Central Opercular Cortex (Left)	3.31	*	*	
73	Temporal Occipital Fusiform Cortex (Left)	76	Frontal Operculum Cortex (Right)	3.25	*	*	
75	Occipital Fusiform Gyrus (Left)	76	Frontal Operculum Cortex (Right)	3.20	*	*	
76	Frontal Operculum Cortex (Right)	88	Supracalcarine Cortex (Right)	3.02	*	*	

Supplementary Figures

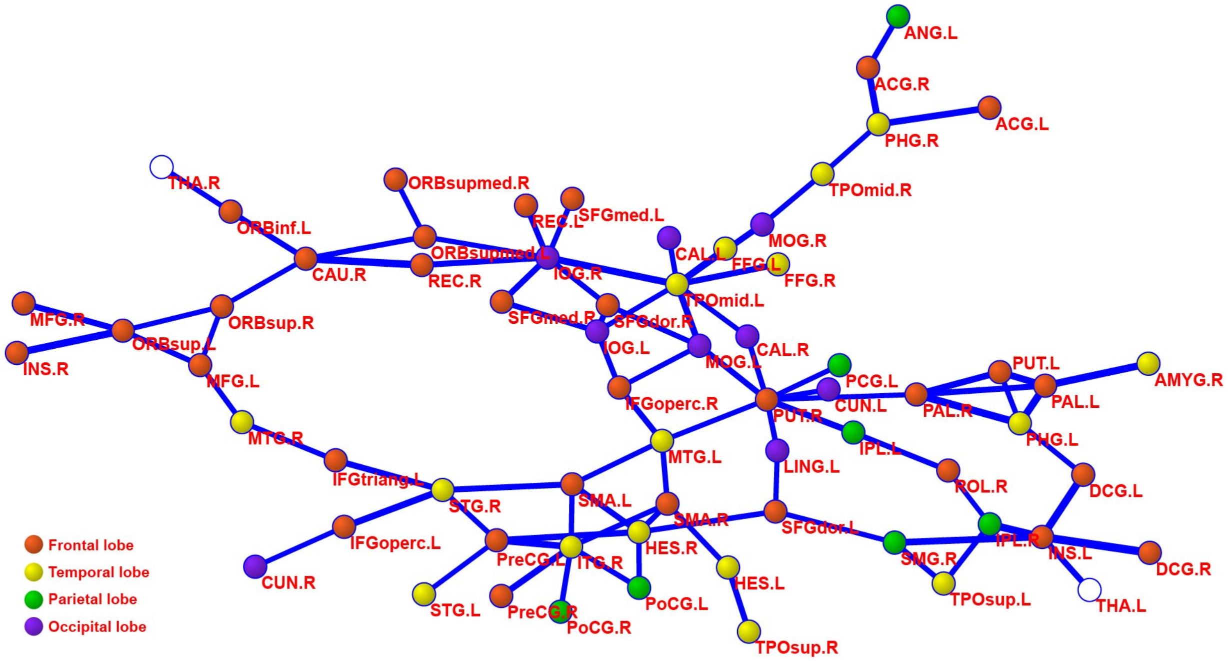

Figure S1. Network component associated with age (p<0.01, regions from the Anatomical Automatic Labeling atlas).

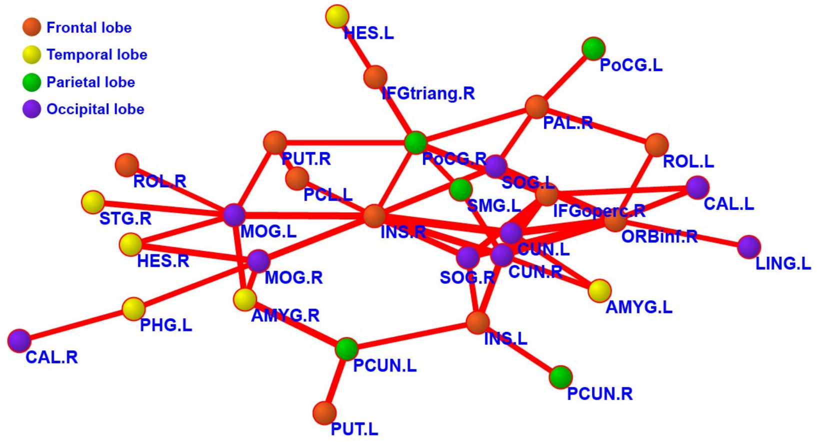

Figure S2. Network component associated with social network embeddedness (continuous) (p<0.01, regions from the Anatomical Automatic Labeling atlas).

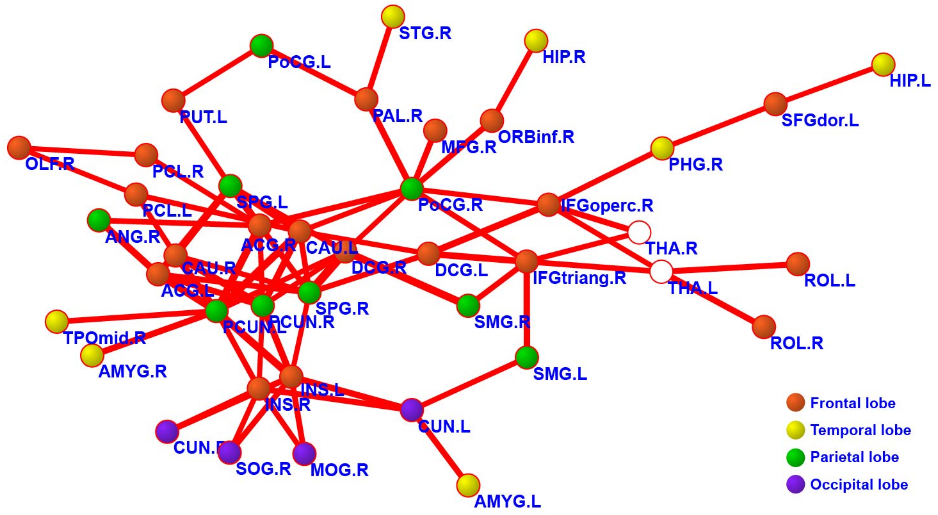

Figure S3. Network component associated with social network embeddedness (low=0, high=1) (p<0.01, regions from the Anatomical Automatic Labeling atlas).

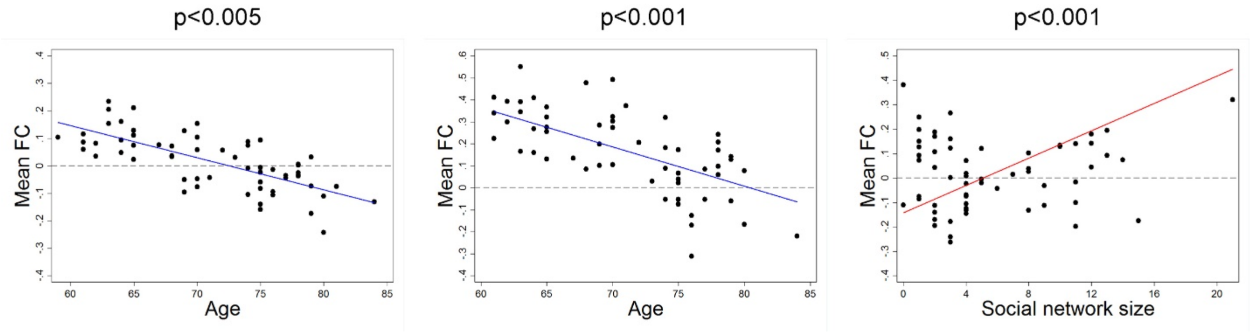

Figure S4. Component-level associations between functional connectivity and age/social network size. Outliers (|DFBETA|>0.25) were excluded (n=61, 59, and 61, respectively) (regions from the Anatomical Automatic Labeling atlas).

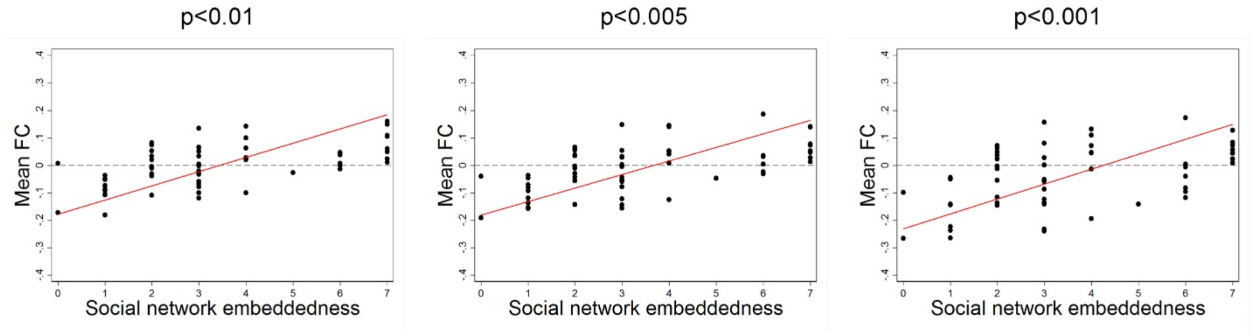

Figure S5. Component-level association between functional connectivity and social network embeddedness (continuous). Outliers (|DFBETA|>0.25) were excluded (n=58, 60, and 58, respectively) (regions from the Anatomical Automatic Labeling atlas).

(a) Age

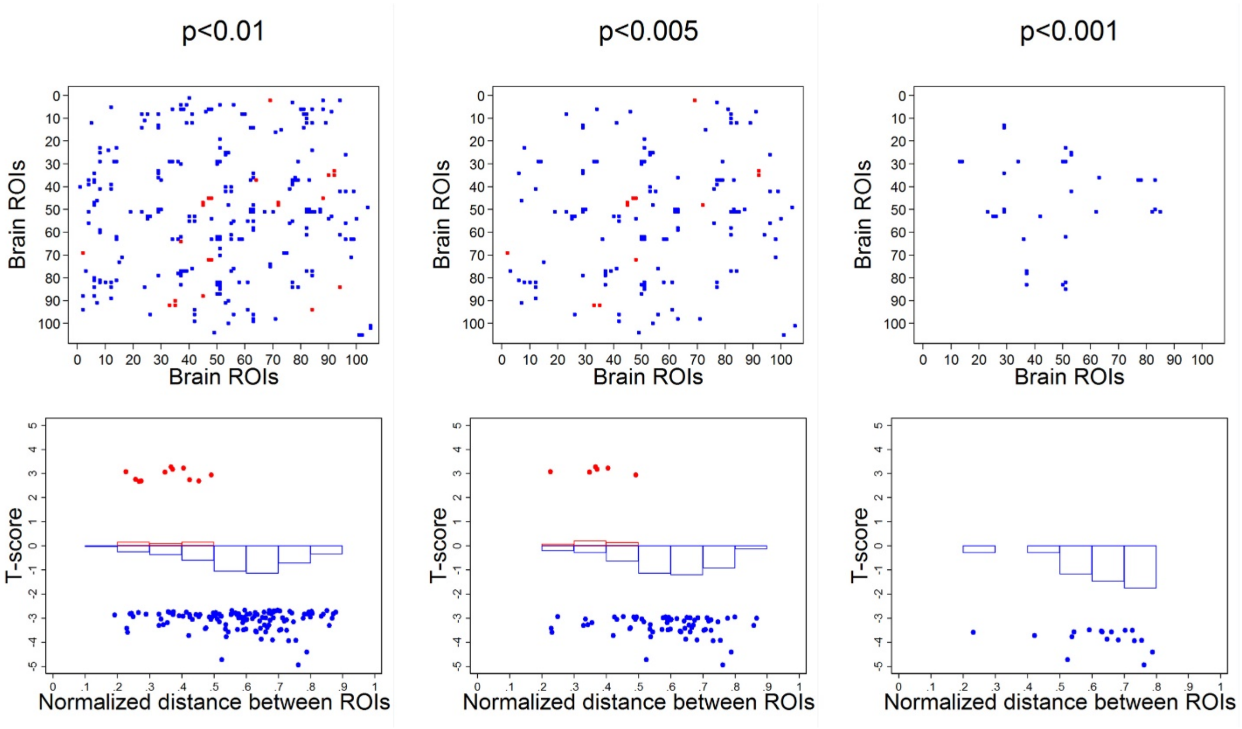

(b) Years of education

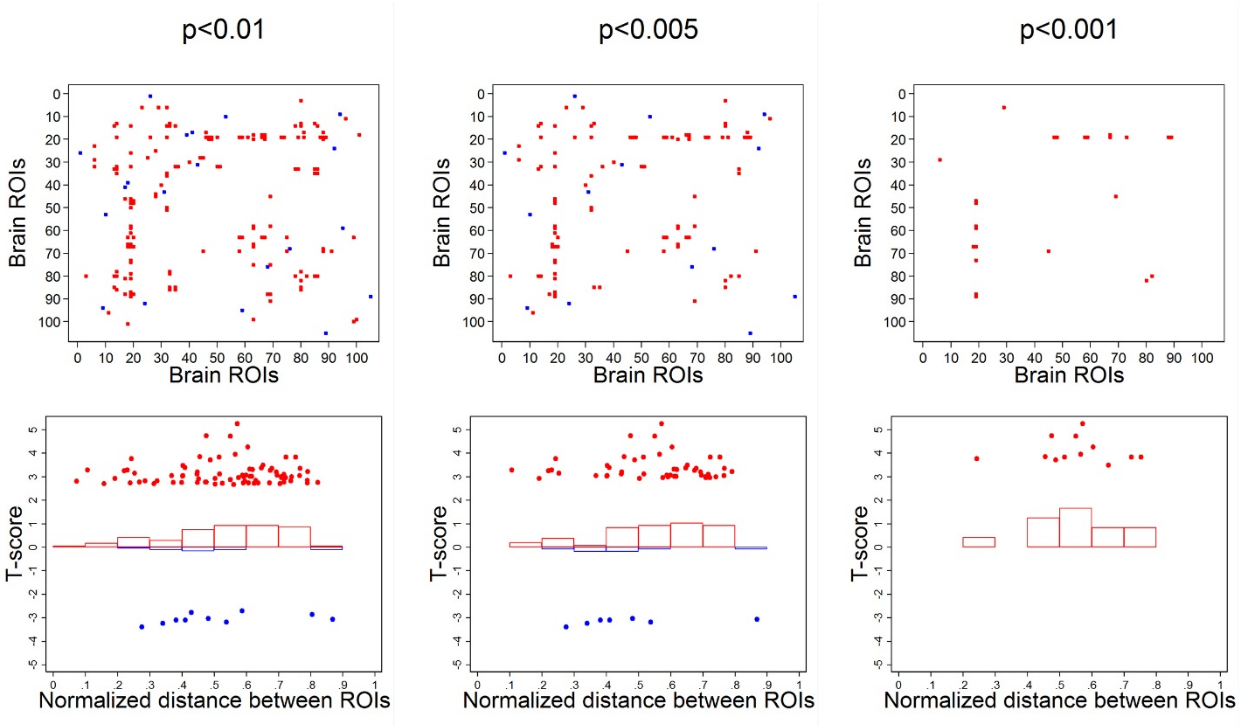

(c) Social network size

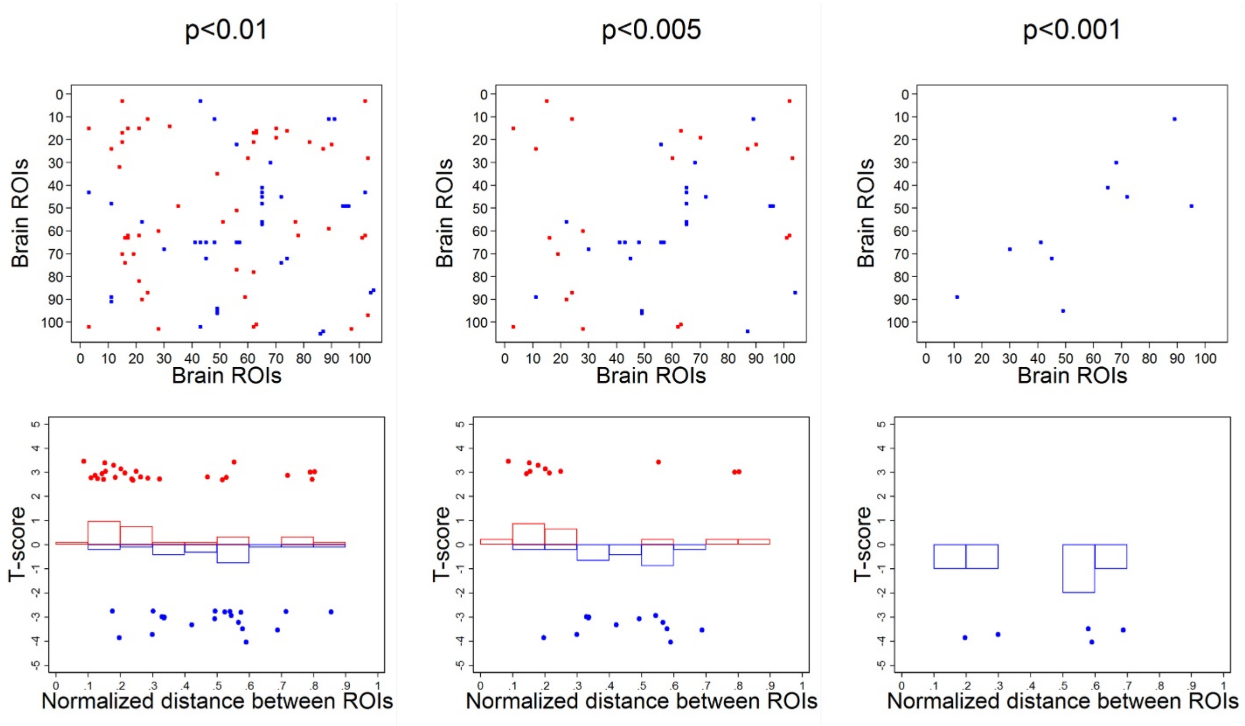

(d) Social network embeddedness (continuous)

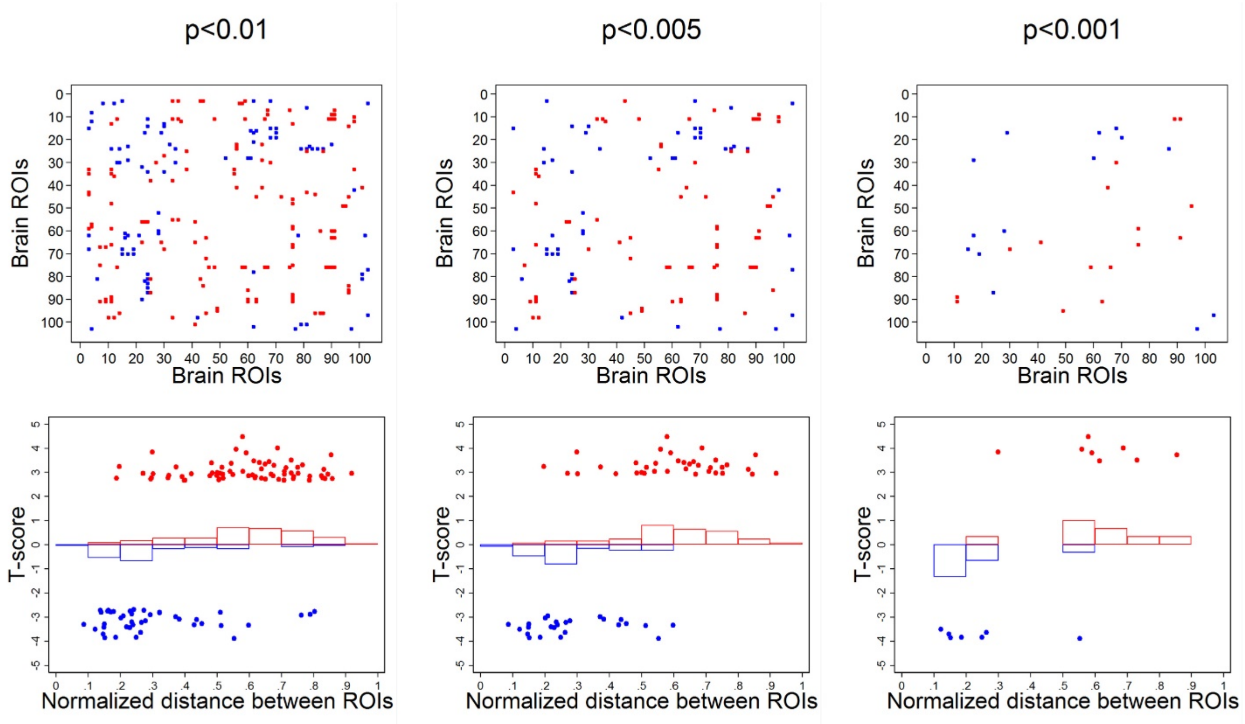

(e) Social network embeddedness (low=0, high=1)

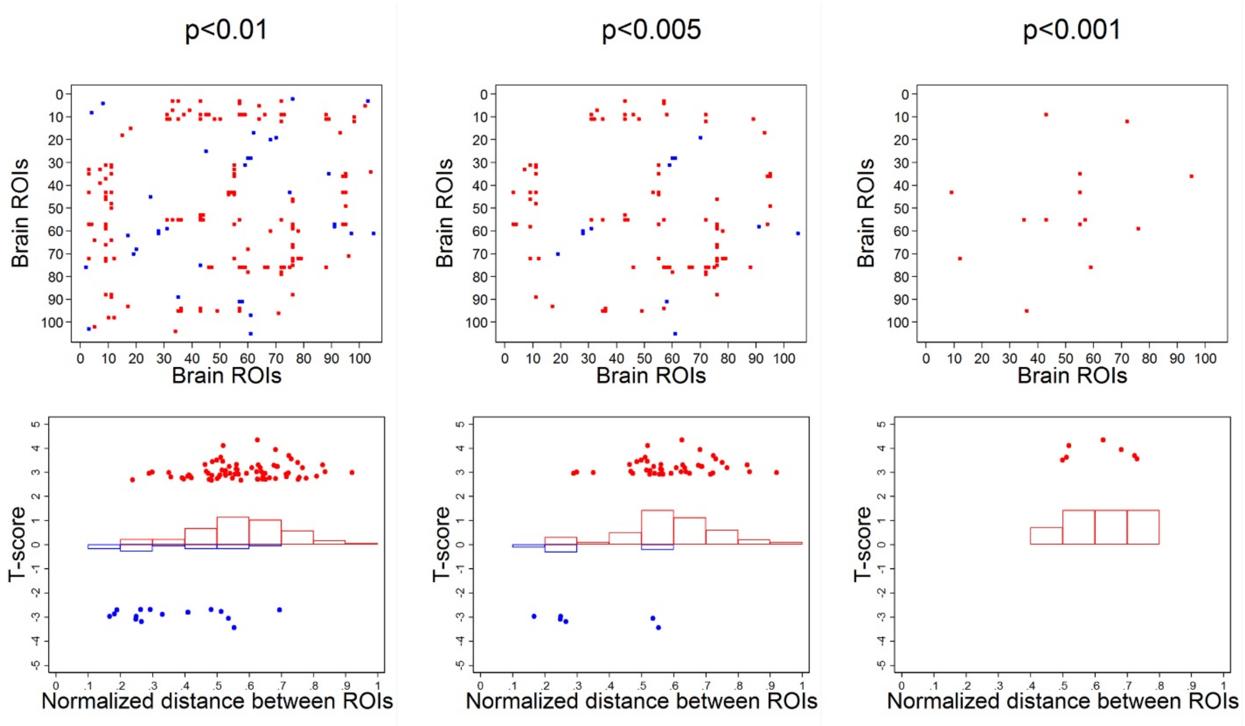

Figure S6. Pair-level associations between functional connectivity and individual characteristics (regions from the Harvard-Oxford probabilistic atlas).

(a) Age

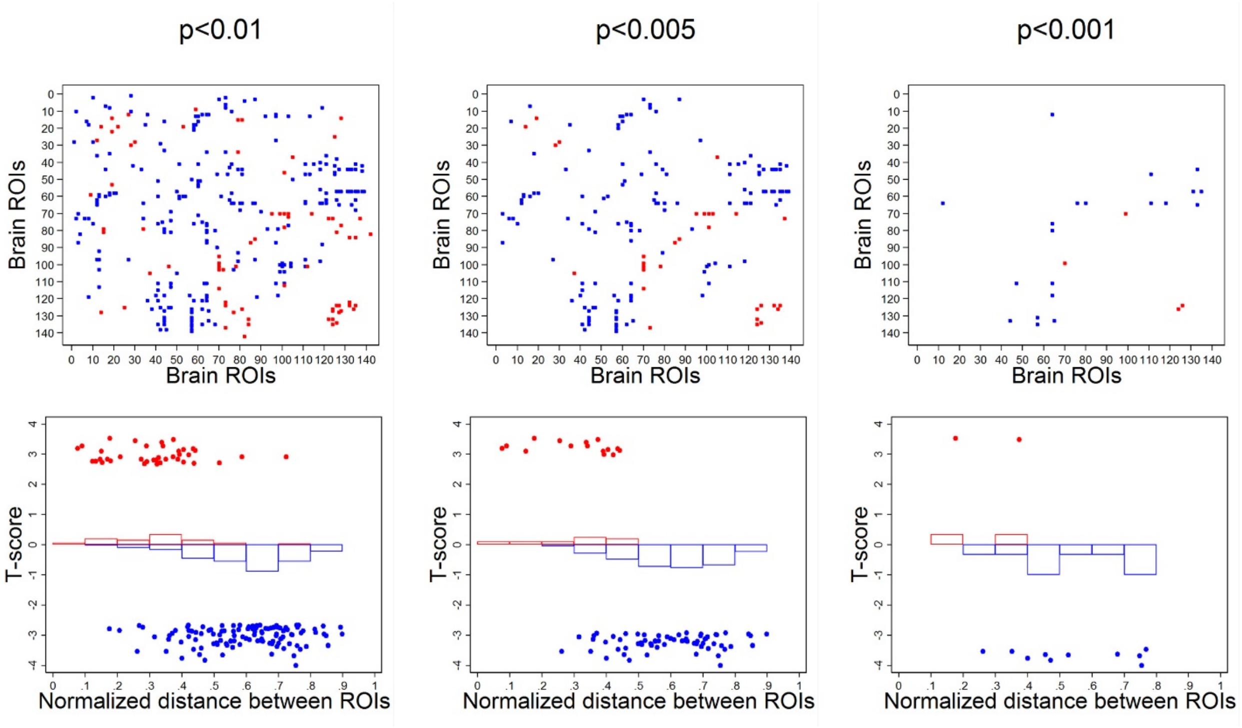

(b) Years of education

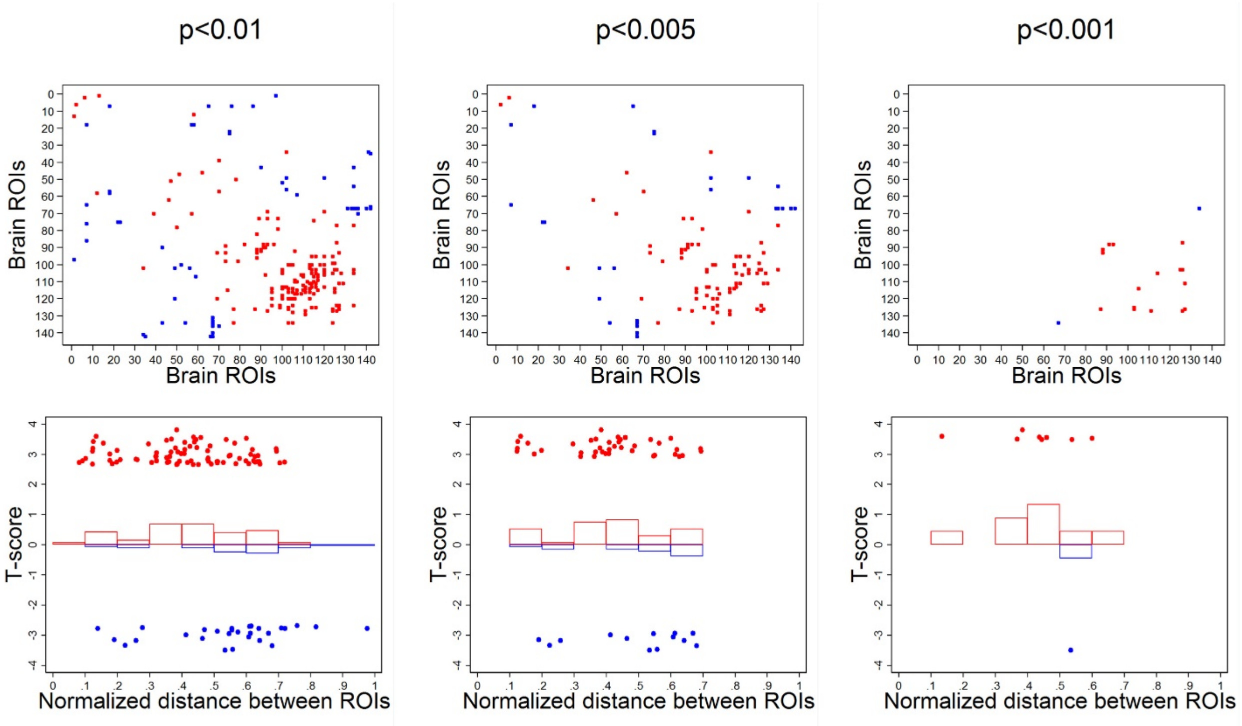

(c) Social network size

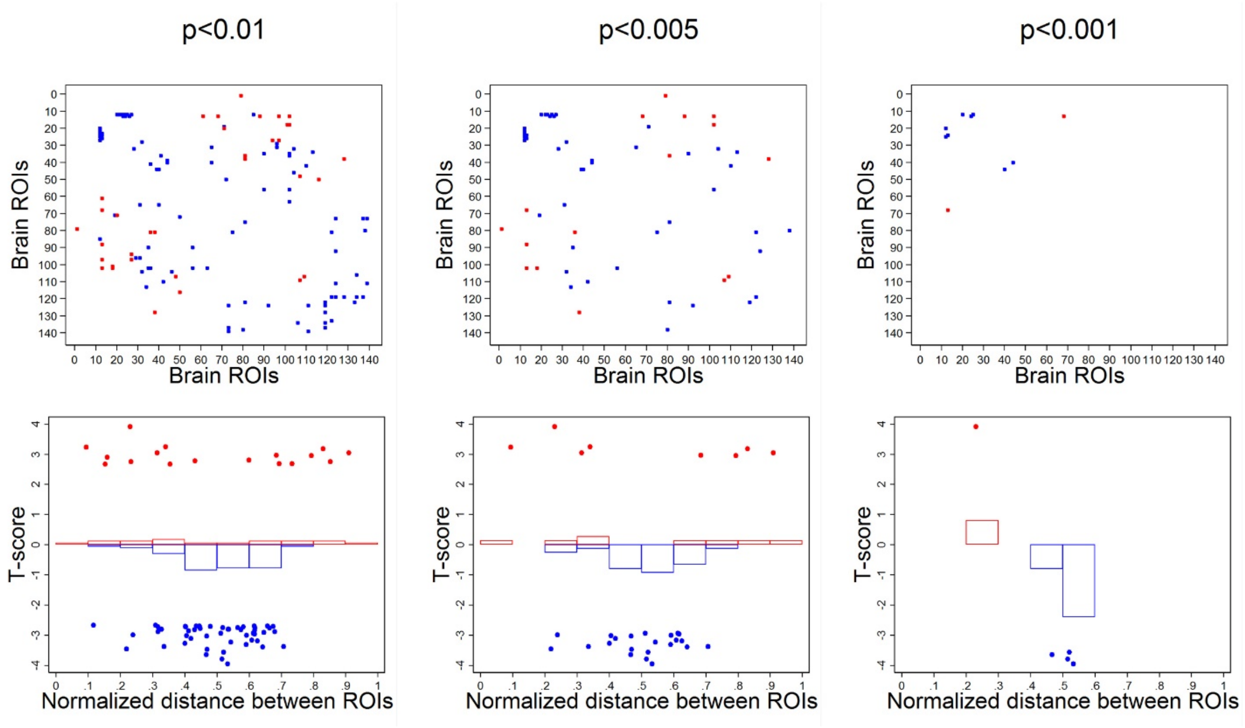

(d) Social network embeddedness (continuous)

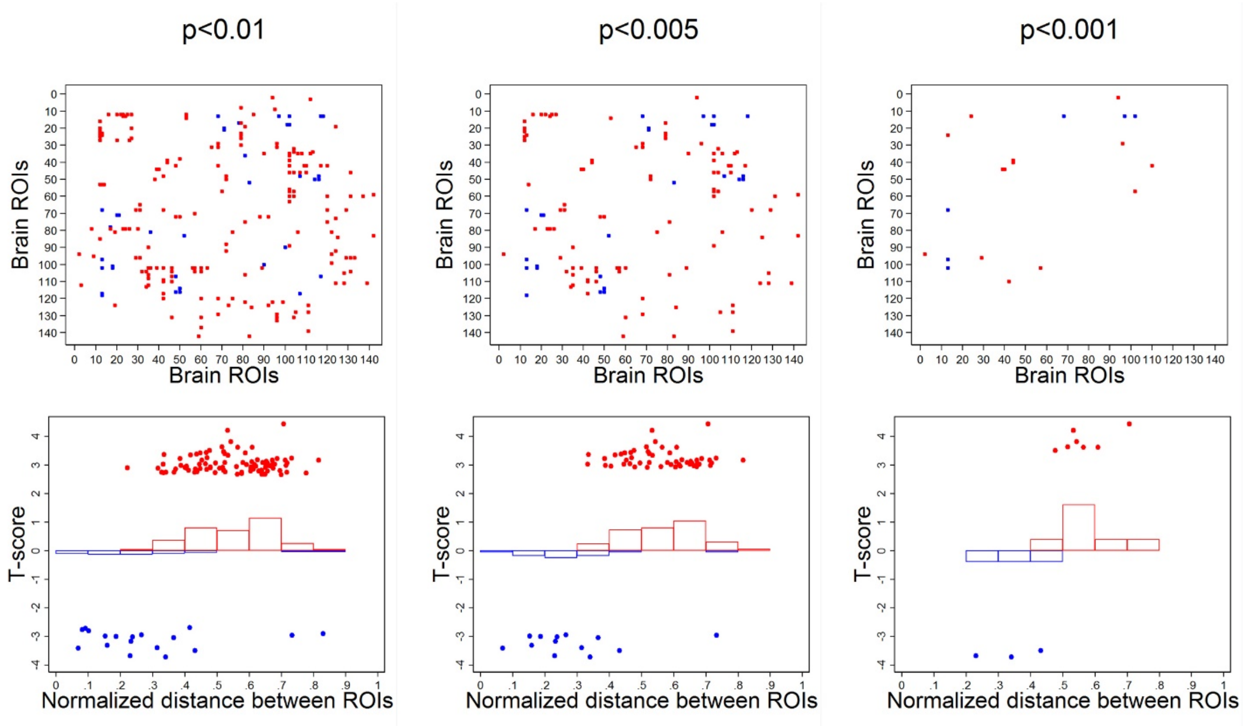

(e) Social network embeddedness (low=0, high=1)

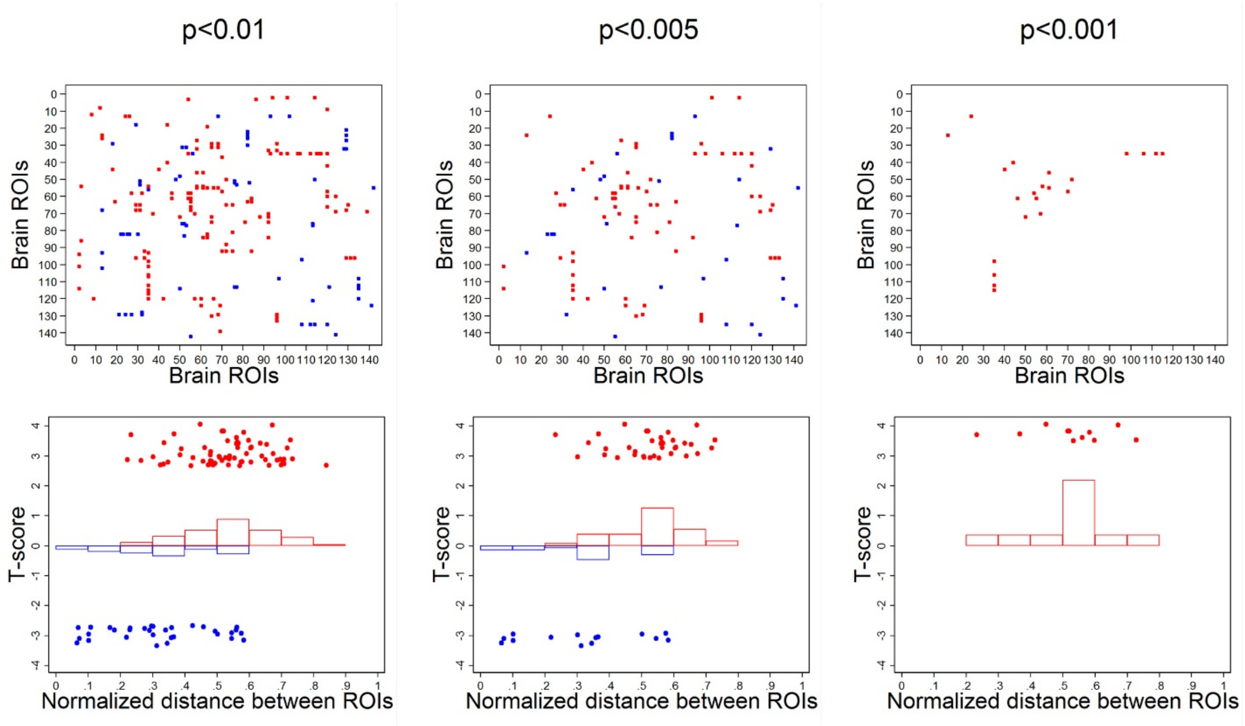

Figure S7. Pair-level associations between functional connectivity and individual characteristics (regions from the Dosenbach atlas).

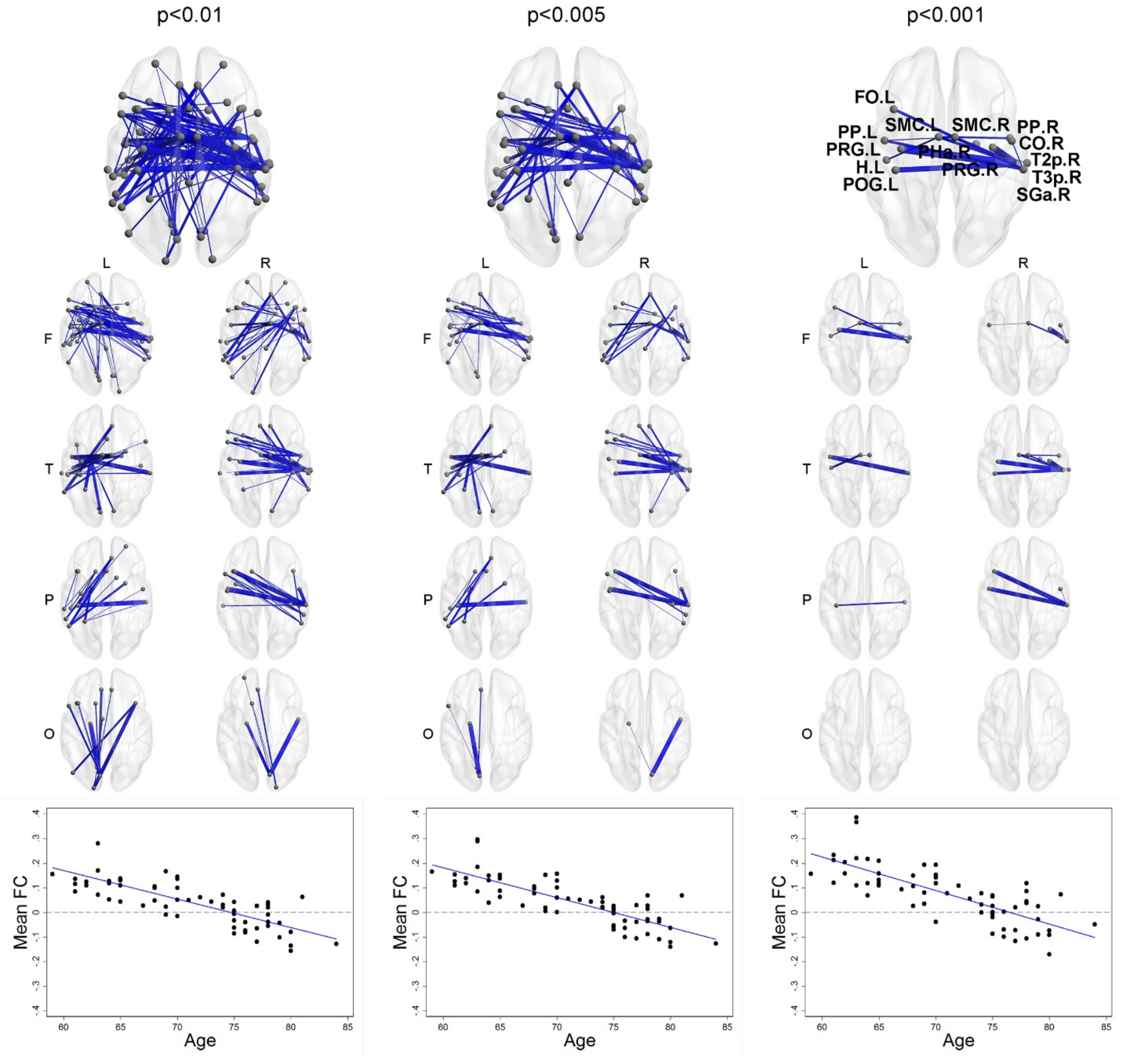

Figure S8. Component-level associations between functional connectivity and age (regions from the Harvard-Oxford probabilistic atlas).

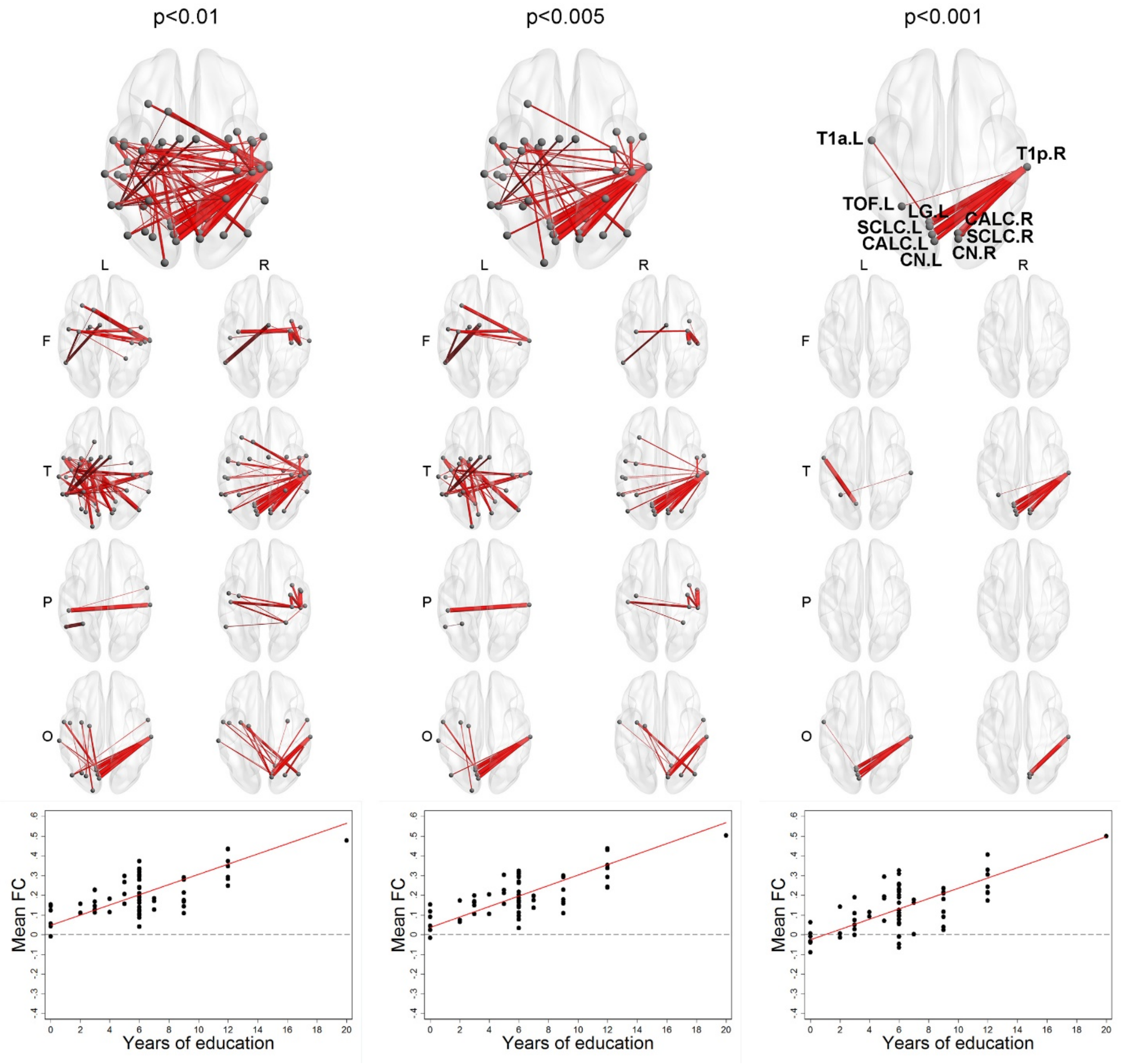

Figure S9. Component-level associations between functional connectivity and years of education (regions from the Harvard-Oxford probabilistic atlas).

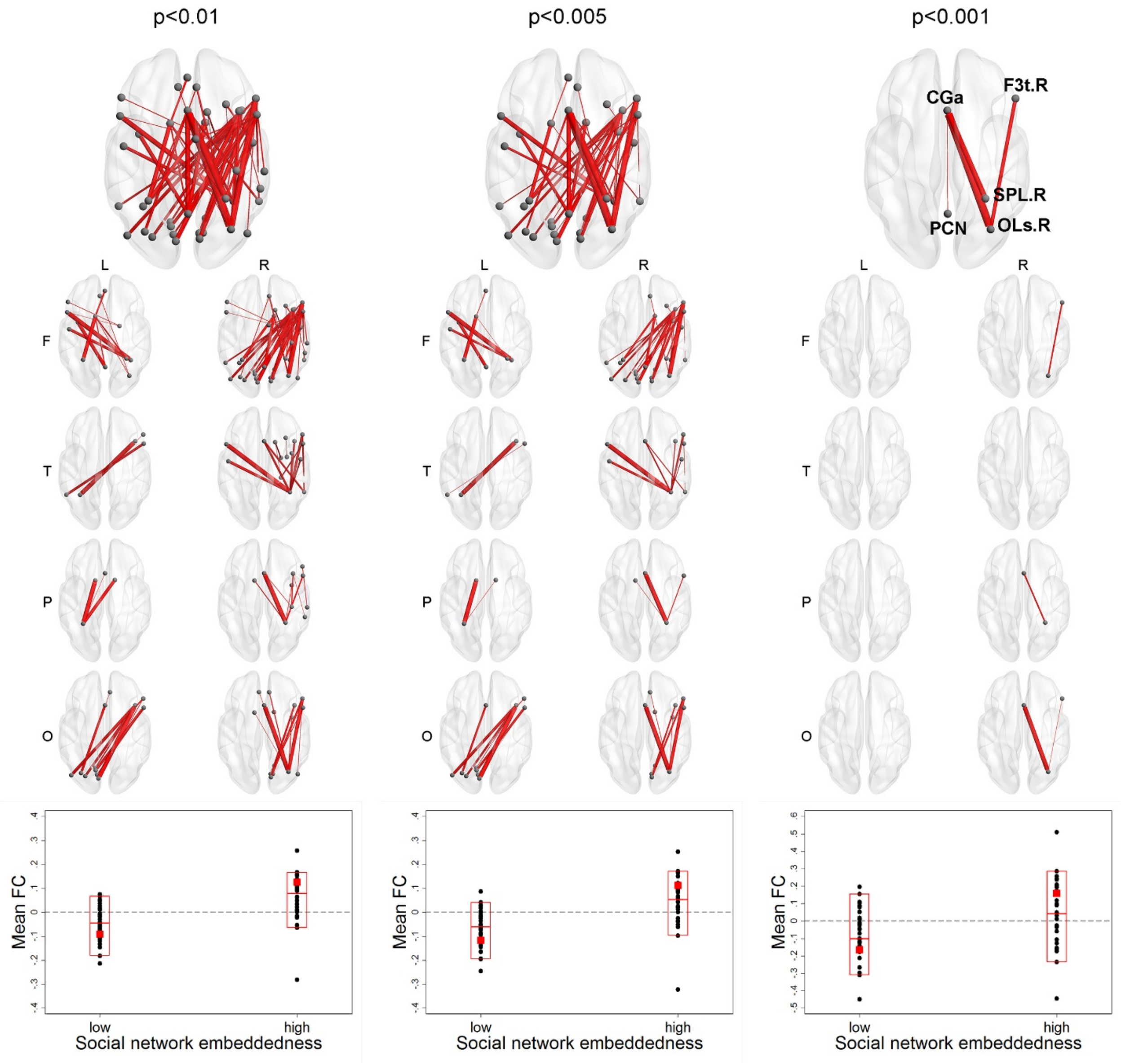

Figure S10. Component-level associations between functional connectivity and social network embeddedness (low=0, high=1) (regions from the Harvard-Oxford probabilistic atlas).

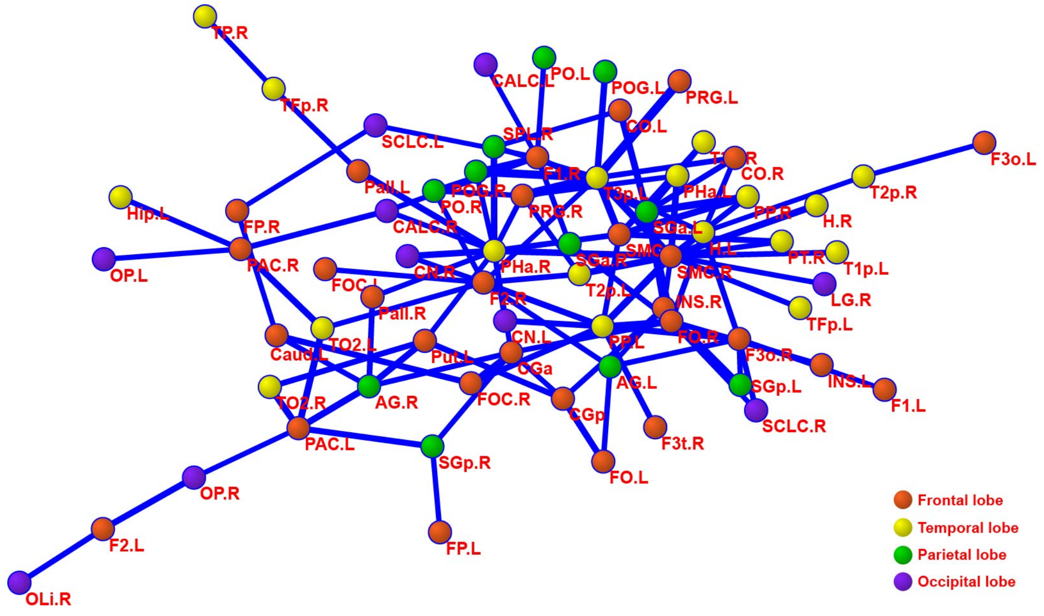

Figure S11. Network component associated with age (p<0.01, regions from the Harvard-Oxford probabilistic atlas).

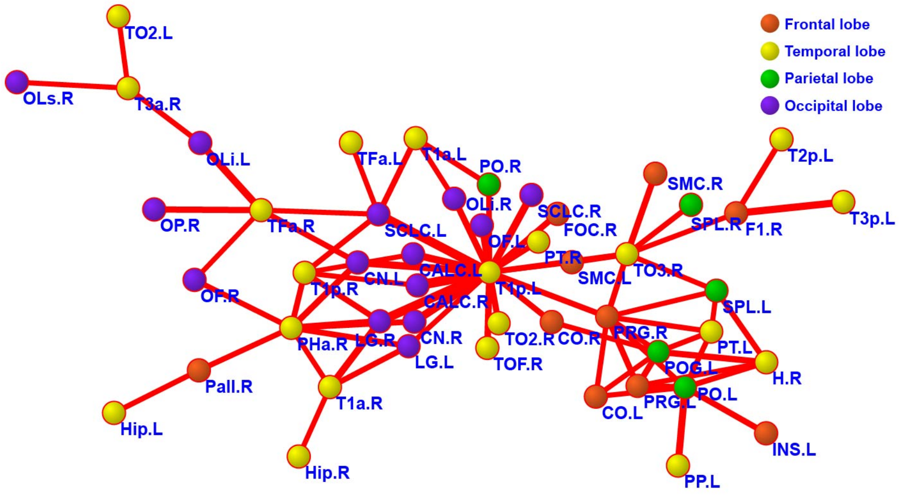

Figure S12. Network component associated with years of education (p<0.01, regions from the Harvard-Oxford probabilistic atlas).

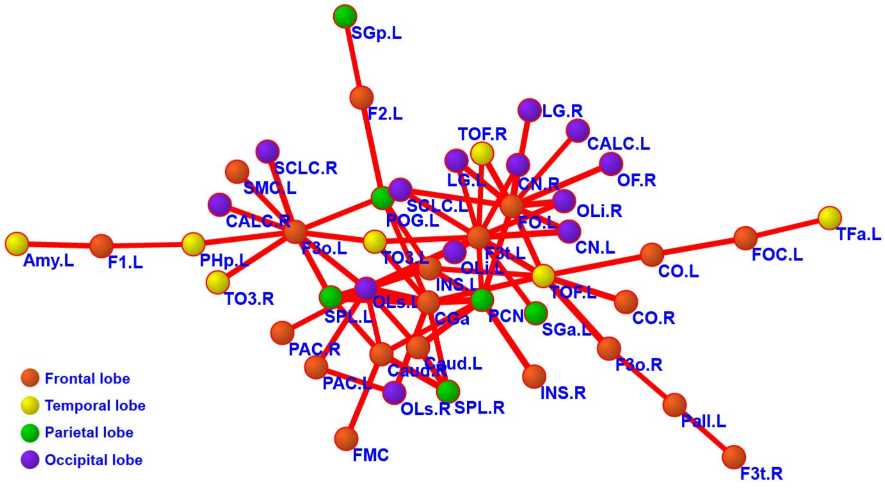

Figure S13. Network component associated with social network embeddedness (low=0, high=1) (p<0.01, regions from the Harvard-Oxford probabilistic atlas).
